# Randomized controlled trial of sulforaphane and metabolite discovery in children with Autism Spectrum Disorder

**DOI:** 10.1186/s13229-021-00447-5

**Published:** 2021-05-25

**Authors:** Andrew W. Zimmerman, Kanwaljit Singh, Susan L. Connors, Hua Liu, Anita A. Panjwani, Li-Ching Lee, Eileen Diggins, Ann Foley, Stepan Melnyk, Indrapal N. Singh, S. Jill James, Richard E. Frye, Jed W. Fahey

**Affiliations:** 1 Departments of Pediatrics, Neurology and Psychiatry, University of Massachusetts Medical School, 55 N. Lake Ave., Worcester, MA 01655 USA; 2 Department of Pharmacology and Molecular Sciences, and The Cullman Chemoprotection Center, Johns Hopkins University School of Medicine, 725 N. Wolfe St., Baltimore, MD 21205 USA; 3 Department of Psychiatry and Behavioral Sciences, and iMIND Hopkins, Johns Hopkins University School of Medicine, 600 N. Wolfe St., Baltimore, MD 21287 USA; 4 Department of Psychological Sciences, Purdue University, 703 3rd St., West Lafayette, IN 47907 USA; 5 Department of Epidemiology, Johns Hopkins Bloomberg School of Public Health, 615 N. Wolfe St., Baltimore, MD 21205 USA; 6 Department of Pediatrics, University of Arkansas for Medical Sciences, 4301 W. Markham St., Little Rock, AR 72205 USA; 7 Barrow Neurologic Institute at Phoenix Children’s Hospital and Department of Child Health, University of Arizona College of Medicine – Phoenix, 475 N. 5th St., Phoenix, AZ 85004 USA; 8 Department of Medicine, Division of Clinical Pharmacology, Johns Hopkins University School of Medicine, 600 N. Wolfe St., Baltimore, MD 21287 USA

**Keywords:** Sulforaphane, Clinical trial, Autism spectrum disorder (ASD), Biomarkers, Placebo effects

## Abstract

**Background:**

Sulforaphane (SF), an isothiocyanate in broccoli, has potential benefits relevant to autism spectrum disorder (ASD) through its effects on several metabolic and immunologic pathways. Previous clinical trials of oral SF demonstrated positive clinical effects on behavior in young men and changes in urinary metabolomics in children with ASD.

**Methods:**

We conducted a 15-week randomized parallel double-blind placebo-controlled clinical trial with 15-week open-label treatment and 6-week no-treatment extensions in 57 children, ages 3–12 years, with ASD over 36 weeks. Twenty-eight were assigned SF and 29 received placebo (PL). Clinical effects, safety and tolerability of SF were measured as were biomarkers to elucidate mechanisms of action of SF in ASD.

**Results:**

Data from 22 children taking SF and 23 on PL were analyzed. Treatment effects on the primary outcome measure, the Ohio Autism Clinical Impressions Scale (OACIS), in the general level of autism were not significant between SF and PL groups at 7 and 15 weeks. The effect sizes on the OACIS were non-statistically significant but positive, suggesting a possible trend toward greater improvement in those on treatment with SF (Cohen’s *d* 0.21; 95% CI − 0.46, 0.88 and 0.10; 95% CI − 0.52, 0.72, respectively). Both groups improved in all subscales when on SF during the open-label phase. Caregiver ratings on secondary outcome measures improved significantly on the Aberrant Behavior Checklist (ABC) at 15 weeks (Cohen’s *d* − 0.96; 95% CI − 1.73, − 0.15), but not on the Social Responsiveness Scale-2 (SRS-2). Ratings on the ABC and SRS-2 improved with a non-randomized analysis of the length of exposure to SF, compared to the pre-treatment baseline (*p* < 0.001). There were significant changes with SF compared to PL in biomarkers of glutathione redox status, mitochondrial respiration, inflammatory markers and heat shock proteins. Clinical laboratory studies confirmed product safety. SF was very well tolerated and side effects of treatment, none serious, included rare insomnia, irritability and intolerance of the taste and smell.

**Limitations:**

The sample size was limited to 45 children with ASD and we did not impute missing data. We were unable to document significant changes in clinical assessments during clinical visits in those taking SF compared to PL. The clinical results were confounded by placebo effects during the open-label phase.

**Conclusions:**

SF led to small yet non-statistically significant changes in the total and all subscale scores of the primary outcome measure, while for secondary outcome measures, caregivers’ assessments of children taking SF showed statistically significant improvements compared to those taking PL on the ABC but not the SRS-2. Clinical effects of SF were less notable in children compared to our previous trial of a SF-rich preparation in young men with ASD. Several of the effects of SF on biomarkers correlated to clinical improvements. SF was very well tolerated and safe and effective based on our secondary clinical measures.

*Trial registration*: This study was prospectively registered at clinicaltrials.gov (NCT02561481) on September 28, 2015. Funding was provided by the U.S. Department of Defense.

**Supplementary Information:**

The online version contains supplementary material available at 10.1186/s13229-021-00447-5.

## Background

Clinical heterogeneity and genetic diversity in autism spectrum disorder (ASD) support approaches to treatment from the perspective of key biochemical pathways [1, 2]. Biomarkers within pathways of hypothetical importance in ASD suggest treatment approaches to ASD that are not directly linked to gene targets may be beneficial [3]. Sulforaphane (SF), an isothiocyanate from broccoli, is a multifunctional phytochemical that has several demonstrated benefits on cellular processes relevant to ASD, including cytoprotective, antioxidant and anti-inflammatory responses, mitochondrial and synaptic function, neuroinflammation and neuroprotective mechanisms, as previously reviewed [4, 5].

We previously reported consistent improvements on clinical measures in treatment of young men with ASD using a SF-rich preparation compared to PL [6]. Benefits persisted with continued treatment in a 3-year follow-up study [7]. Positive clinical effects as well as findings in urinary metabolites were also reported in an open-label trial in children and young adults using the same glucoraphanin (GR)-rich extract of broccoli seeds with added myrosinase that we used in this study [8]. A recent clinical trial of SF in combination with risperidone demonstrated greater improvements in irritability, hyperactivity and noncompliance, compared to risperidone alone, in children with ASD [9].

**Fig. 1:**
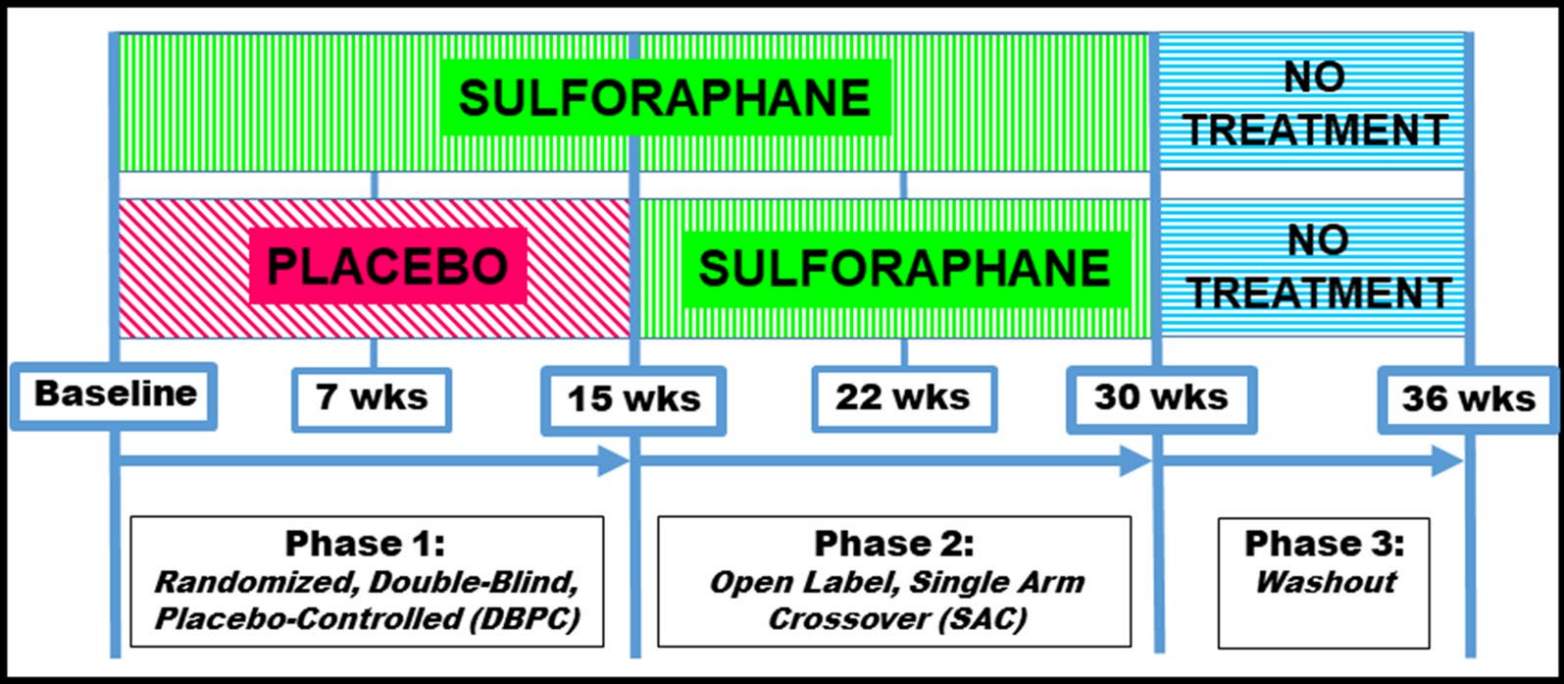
Study timeline (6 total visits); in Phase 1 (1–15 weeks) participants were randomly assigned to either SF or placebo with visits at baseline, 7 and 15 weeks. In Phase 2 (16–30 weeks), all participants received SF and returned for follow-up at 22 and 30 weeks. In Phase 3 (31–36 weeks), there was no treatment, with a final visit at 36 weeks

This report describes the results of our 36-week randomized parallel double-blind placebo-controlled, phase 2 clinical trial of oral GR, the stable metabolic precursor of SF from a GR-rich extract of broccoli seeds, and myrosinase, the enzyme that converts GR to SF (hereafter referred to as “SF”), in 45 children with ASD, ages 3–12 years (Fig. 1). Our aims were to determine the effects of SF on social responsiveness and problem behaviors in boys and girls with ASD, to assess its safety and tolerability and to evaluate cellular biomarkers that support hypothesized mechanisms of action of SF in ASD.

For our primary outcome measure we used the Ohio Autism Clinical Global Impressions Scale (OACIS) [10, 11], and for secondary outcome measures, caregivers completed the Social Responsiveness Scale-2 (SRS-2) [12] and the Aberrant Behavior Checklist (ABC) [13].

To determine biochemical effects of SF in peripheral blood mononuclear cells (PBMC; which was considered to be the most accessible body compartment for these analyses), and based on the results of our pilot study [5], we chose to examine cytoprotective gene products regulated by nuclear factor erythroid 2-related factor 2 (Nrf2), the master regulator of cellular redox homeostasis and an inhibitor of a key pro-inflammatory pathway [14], of which both functions are critical factors in the neuropathology of ASD. These Nrf2-dependent enzymes are NAD(P)H:quinone oxidoreductase-1 (NQO1), heme oxygenase 1 (HO-1), an essential enzyme in heme catabolism, and xCT (SLC7A11), a cystine/glutamate antiporter regulated by Nrf2 that imports cystine into the cells while exporting glutamate and preserves intracellular redox balance [15]. We also measured the gene expression of nuclear factor-κB (NF-κB)-regulated inflammatory biomarkers—cyclooxygenase-2 (COX-2), and the cytokines tumor necrosis factor alpha (TNF-α), interleukin 6 (IL-6) and interleukin 1 beta (IL-1β). Our aim was to assess the in vivo effects of SF, which has been shown in cultured rat macrophages in vitro to attenuate the NF-κB pathway [16]. Heat shock proteins (HSP27 and HSP70) were examined because they are upregulated by SF in vitro [17] and are cytoprotective and responsive to fever, which has been found to ameliorate symptoms in some children with ASD [18]. Due to the importance of cellular oxidative stress and mitochondrial function in ASD, we measured free reduced, oxidized and total glutathione (fGSH, fGSSG and tGSH, respectively) and the ratios fGSH/fGSSG and tGSH/fGSSG in plasma, to assess subjects’ instant redox status [19, 20] in response to SF and mitochondrial respiration [21] in response to SF.

## Methods

### Participants

Children ages 3–12 years with ASD were recruited from UMass outpatient clinics (approximately 60%), local and regional autism societies and pediatricians (20%), and 20 percent were self-referred. All visits (one screening and 5 subsequent visits over 36 weeks) took place in the Clinical Research Center or Department of Pediatrics at the UMass Memorial Medical Center in Worcester, MA.

The study was approved by the Institutional Review Boards (IRB) at the U.S. Department of Defense (A-18817.a), UMass Medical School (H00007832) and Johns Hopkins University (00084331), conducted under IND 127062 and registered as NCT02561481. All participants and their caregivers were consented at the screening visit. A Research Monitor and Data Safety Monitoring Board met regularly with the study staff to review progress and safety concerns.

### Screening assessments for eligibility

**Fig. 2:**
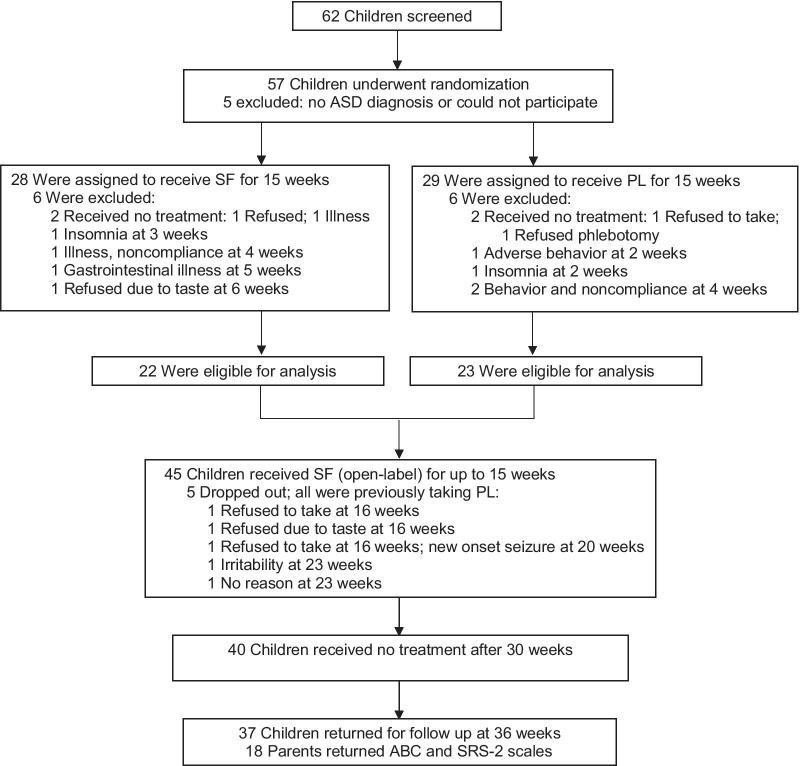
Randomization and follow-up. *SF* sulforaphane and *PL* placebo

Criteria for inclusion were a diagnosis of moderate to severe ASD, ages 3–12 years and a parent or guardian for consent; for exclusion: seizure within 1 year, impaired renal, hepatic or thyroid function, current infection or treatment with antibiotics, medications that may modify the testing of ASD (e.g., prednisone), and chronic medical disorders. Of the 62 children who qualified for the study on initial screening, 5 were excluded because of misdiagnosed ASD or inability to participate (Fig. 2). All screening tests for eligibility were carried out by an experienced examiner (A.F.) using the following measures to determine the diagnosis of ASD along with individual features of cognition and behavior:

The Autism Diagnostic Observation Schedule-2nd Edition (ADOS-2) [22] was administered to confirm the diagnosis of autism or autism spectrum. The ADOS-2 consists of standard, semi-structured activities, chosen based on the language level of the individual that enable the examiner to observe and rate ASD-related behaviors. The ratings are used to determine the classification: autism, autism spectrum, or non-spectrum.

Vineland Adaptive Behavior Scale, Second Edition (VABS-II) [23], was conducted with the participant’s primary caregiver, via a semi-structured interview to measure functioning in communication, daily living and socialization skills.

Leiter-R [24] is a nonverbal test of intelligence and cognitive functions from ages 2 to 20 years. Six subtests that comprise the full-scale intelligence quotient (FSIQ) from the Leiter-R were used to characterize the general nonverbal intelligence of each participant.

The OACIS, our primary outcome measure, is an instrument for rating the severity of symptoms in 10 categories, including the general level of autism, social interaction, aberrant and repetitive behavior, verbal and nonverbal communication, hyperactivity, anxiety, sensory sensitivities and restricted and narrow interests. Each category is rated from 1 (normal) to 7 (most severe). The OACIS-S (severity) was rated at the initial visit, then used as a reference point at follow up visits when the OACIS-I (improvement) was scored.

The Social Responsiveness Scale-2nd Edition (SRS-2), a secondary outcome measure, is a 65-item rating scale measuring deficits in social behaviors in 6 subscales related to ASD [12]. The SRS-2 was completed by consistent caregivers following each visit and returned by mail.

The Aberrant Behavior Checklist (ABC), a secondary outcome measure, is a 58-item behavior rating scale used to measure behavior problems across 5 subscales [13]. The ABC was also completed by caregivers following each visit and returned by mail.

Medical histories of all children were reviewed, including evaluations leading up to the diagnosis of ASD and subsequent therapies and treatments. Children’s responses to fever [18] and developmental regression were recorded, based on reported histories from their caregivers.

### Dosage and administration

We were unable to produce or obtain SF-rich preparations for this study like those used in our previous study [6]. Based on prior testing of alternative preparations [25–27], we used commercially available crushable tablets containing 125 mg broccoli seed extract as a source of GR (equivalent to 34 µmol GR calculated to yield at least ~ 15 µmol SF), 50 mg broccoli sprout extract (the source of myrosinase, the enzyme that converts GR to SF), 15 mg ascorbic acid, 55.9 mg microcrystalline cellulose and 4.1 mg silicon dioxide. Tablets commercially produced as Avmacol® were provided by Brian Cornblatt, Ph.D.; Nutramax, Edgewood, MD. Placebo tablets, identical in size and similar in appearance to the active tablets, contained microcrystalline cellulose, red, yellow and black coloring and magnesium stearate, and were produced by Dr. Stephen Hoag, University of Maryland Department of Pharmacy. Pill crushers were supplied to all families. Randomization was performed, 1:1 SF and placebo, and tablets were dispensed by the Investigational Drug Service at UMass Memorial Medical Center (Natasha Sanil, MS, RPh and staff). The participants, families and caregivers, as well as all research staff and monitors, were blinded to group assignment throughout the trial. Unblinding for all participants took place at the end of the trial, except for two participants, as described below under Results.

In a 2-week pilot study of 10 boys (6–12 years; not included in the main study) with ASD, oral GR and myrosinase (equivalent to 2.2 μmol/kg/day SF) demonstrated bioavailability of 47.0% ± 13% (range 29.4–68.4%) of GR dose [5], close to 50% that we anticipated from previous work in adults with GR plus myrosinase [26]. Details of the pilot study can be found in [5].

### Study design (Fig. 1)

After screening, 57 children were assigned by the Research Pharmacy, double-blinded, and randomized 1:1, to either treatment with SF or identical placebo for 15 weeks (Phase 1), then open-label treatment of all children with SF from 16 to 30 weeks (Phase 2), followed by no treatment from 31 to 36 weeks (Phase 3). Six clinical visits were scheduled: Screening (baseline, time 0), 7, 15, 22, 30 and 36 weeks. Screening included ADOS-2, Vineland, and Leiter scales, and the OACIS-S. Participants were assessed by senior clinicians (A.W.Z. or S.L.C.) with physical examination at each visit along with assessment using the OACIS-I [11] at visits 2 through 6. Parents or consistent caregivers experienced with ASD completed the ABC and SRS-2 after all visits and returned them by mail. Blood and urine specimens were collected at each visit for routine clinical laboratory studies as well as biomarker analysis.

SF was administered as GR-rich broccoli seed extract tablets containing myrosinase as described above, in an approximate dosage equivalent to 1 µmol SF/lb/day (2.2 µmol/kg/day) body weight. This dosage is roughly equivalent to the dosage that was used in our previous clinical trial of SF in male adolescents and adults with autism [6]. The total dose per day was based on the study participants’ body weight: 30–50 lbs, 3 tablets (45 µmol/day); 50–70 lbs, 4 tablets (60 µmol/day); 70–90 lbs, 6 tablets (90 µmol/day); 90–110 lbs, 7 tablets (105 µmol/day); 110–130 lb, 8 tablets (120 µmol/day). All caregivers were given a manual pill crusher, and if a child was unable to swallow tablets, parents were asked to crush the tablets and mix the contents into small cups of applesauce or other food, without heating. Placebo tablets in equal number were used based on body weight. The study drug (SF or placebo) was administered orally, at approximately the same time, once a day (usually in the morning to avoid insomnia), and preferably avoiding taking it with a heavy meal. Caregivers were requested to keep daily medication diaries. All children received a toy and gift card for $15 at each visit. Compliance was measured by residual pill counts, medication diaries and plasma levels of SF metabolites.

Two phone visits with caregivers took place between visits 1 and 2, and 3 and 4, to review the children’s progress and answer questions. Participants’ other concomitant medications, including psychopharmaceuticals, vitamins and dietary supplements were recorded and could be continued. Caregivers were asked to inform the study staff when changes were made to their children’s medications. Blood and urine specimens were collected at each visit for complete blood count (CBC), comprehensive metabolic profile (CMP; including serum glucose, creatinine, blood urea nitrogen, AST, ALT), thyroid stimulating hormone (TSH) and urinalysis.

### Biomarker analysis

Samples for biomarkers were obtained from study participants at each visit. Eight mL of whole blood was drawn into Vacutainer CPT tubes (Becton, Dickinson and Company, Franklin Lakes, NJ, USA) at room temperature and processed for PBMC isolation [5]. After centrifugation, plasma was quickly frozen for cyclocondensation and oxidative stress markers. PBMC pellets were washed twice with PBS and were stored at − 80 °C for future RNA isolation.

Sulforaphane and other isothiocyanates (ITC) are conjugated by glutathione (GSH) which then undergoes further enzymatic modifications to give rise sequentially to the cysteinylglycine–, cysteine– and N-acetylcysteine–ITC conjugates, all of which are dithiocarbamates (DTC) and are detected in the cyclocondensation reaction-HPLC assay as described previously [28].

Total cellular RNA was isolated from PBMCs and complementary DNAs were synthesized as previously described [14]. Quantitative real-time PCR analysis was performed using the Applied Biosystems QuantStudio™ 3 Real-Time PCR System (Thermo Fisher Scientific, Waltham, MA, USA). Primer sequences for gene amplification are shown in Additional file 1: Table S1A. Relative mRNA expression was normalized to GAPDH. Gene expression was calculated using the comparative 2^−ΔΔCT^ method [29].

*Sample preparation for measurement of plasma total glutathione (tGSH), plasma free reduced glutathione (fGSH) and oxidized glutathione disulfide (fGSSG)* For detailed procedures see Additional file 1: Table S1B, S1C and [30].

*Measurement of mitochondrial function* For detailed procedures see Additional file 1: Table S1D and [21].

### Statistical analysis

All analyses were completed using Stata v16 (StataCorp LLC) and MS Excel 2016 (Microsoft Corp.). Our hypothesis was that SF would be superior to PL for 15 weeks with respect to improvement in clinical features of ASD and its effects on selected biomarkers. We planned to enroll a total of 50 participants in the study, randomized 1:1 to SF and PL, which a priori had more than enough power to reject the null hypothesis (i.e., no significant difference in the primary outcome measure (OACIS-I) average score at 15 weeks between two treatments). Forty subjects (20 on SF and 20 on PL) entering this two-treatment parallel-design study would have provided at least 80% power to test the primary hypothesis for the OACIS-I score using a two-tailed two-sample *t* test with *α* = 0.05 and assuming that the true difference in average change in OACIS-I was 4 units with a SD of 4.9 units.

Data were evaluated from 45 children who completed 15 weeks of treatment in Phase 1 (Fig. 2). While intention to treat (ITT) analysis was used, missing data were not imputed due to unpredictability and the small sample size.

The OACIS-I general score and subscale values were recoded to follow a natural numerical order, in which 4 (no change) was recoded as 0; following this scale, scores of 3–1 were recoded as + 1 to + 3, respectively, to denote improvement, and scores 5–7 were recoded as − 1 to − 3, respectively, for worsening. Half-point values were coded accordingly.

The OACIS-I, SRS-2, and ABC total scores and subscale scores, along with all the biomarkers, were treated as continuous variables. For each of these outcomes, we reported descriptive analyses at each time point. Three ABC subscales (lethargy, irritability and stereotypy) were square root transformed and all fold-changes of biomarkers were natural log transformed to fit a normal distribution assumption. For all the scales and biomarkers, means at each visit by intervention group took into consideration matched pairs (matched on sex and fever response). Paired t-tests were used to compare means of SF group versus PL group changes from baseline at each visit. OACIS-I results were expressed as change from baseline scores (OACIS-S). Due to this analytical methodology, only pairs with complete data for each time point were included in the analysis for that time point. Standardized mean difference and precision estimates were also calculated for SF group vs PL group for OACIS-I scores at each visit using Cohen’s *d* to report effect sizes.

### Length of exposure analysis

This analysis of SRS-2 and ABC data considered only the periods when both groups (SF and PL) had exposure to SF treatment, with the PL group exposed only during the open-label phase. Thus, the dataset for 7 weeks of exposure for this analysis comprised data from 0 to 7 weeks for the SF group and 15 to 22 weeks for the (former) PL group when taking SF; 15 weeks of exposure for this analysis comprised data from 0 to 15 weeks for the SF group and from 16 to 30 weeks for the (former) PL group when taking SF, combined as one large group. The data for 0–22 and 0–30 weeks of exposure were solely reflective of the SF group, as only these individuals were exposed to SF for this length of time. The sample size for these weeks was thus also halved. This analysis was non-randomized as it did not consider a PL control, and should be interpreted accordingly. The length of exposure analysis was also conducted stratifying by fever effect and developmental regression to further explore these phenomena.

### Regression modeling

Mixed-effects modeling was carried out to account for repeated measures of the outcome measures on the SRS-2 and ABC and their respective sub-scores. In these models, the fixed effects were treatment and time (visit). The fixed effect for treatment shows the difference between treatment groups at the group level. Similarly, the fixed effect for visit relates the difference between visits (compared to baseline at visit 0) at the group level. The random effect was subject-specific (intercept and slope) at each visit to account for within-subject variability. Three models were produced for each outcome measure to compare results between different time points:

- Model 1 includes the treatment variable (SF) and the categorical visit variable including visits 0–30 weeks.
- Model 2 includes the treatment variable (SF) and the categorical visit variable including visits 30–36 weeks to show the effect of the washout period.
- Model 3 includes the treatment variable (SF) and the categorical visit variable including visits 0 and 36 weeks to show the difference between baseline and post-washout.

Because the treatment (SF) and visit variables adjust for each other in the model, the beta coefficient for visit number accounts for treatment and the beta coefficient for treatment are irrespective of visit number.

We chose not to adjust for multiple comparisons as this study was the first to examine this treatment among children with ASD in this age range. As such, we regard this study as exploratory and additional trials with larger samples are required to draw any firm conclusions. Further, our analysis methods of mixed modeling and MANOVA each control for experiment-wide type I error rates [31, 32].

### Statistical analysis for biomarkers

Biomarkers were evaluated for their association with SF using simple linear regression at each visit and were expressed as change from baseline. Free reduced GSH, total GSH and GSSG were natural log transformed. There were no significant differences between the two arms for any of the potential confounders at baseline. Therefore, none were included in any of the models. Because hemolysis can cause release of red cell GSH and artificially increase measured GSH levels, we analyzed the data with and without inclusion of hemolyzed samples.

A nonparametric method (Wilcoxon Rank Test) was used to also compare nine biomarkers across various categories of interest: SF versus placebo, clinical improvement versus no clinical improvement, history of developmental regression versus no regression, history of fever effect versus no fever effect—at baseline, 15 weeks and 30 weeks. Clinical improvement was defined as > 20% decrease in total ABC score compared to baseline for all participants. The boxplots were made using the Wilcoxon ranks and p-values reported when significant (*p* < 0.05).

### Analysis of mitochondrial function

An analysis was conducted using PAWS Statistics 18 (SPSS Inc, Quarry Bay, HK) general linear model module with a within-factor (repeated measure) of time and between subject variable of active treatment with SF versus PL and developmental regression. A covariate of change in total ABC score was used in some models. First, the effect of the drug treatment was examined using the before and after double-blind, placebo-controlled (DBPC) time point to determine if the drug treatment affected any of the respiratory parameters. Second, the change in the respiratory parameters across the open-label phase of the study was examined. Third, the three time points were included in the model with the covariate of change in ABC score from baseline to the end of the open-label trial to determine if changes in respiration across the entire trial correlated with change in ABC score across the entire trial. Both a linear and quadratic relation was investigated between the change in respiratory parameters and change in ABC scores.

## Results

### Study cohort and participants who dropped out

Of the 62 children who qualified for the study on initial screening, five were excluded because of misdiagnosed ASD or inability to participate (Fig. 2). Fifty-seven children were randomized after which 12 did not complete 15 weeks of treatment in Phase 1: four were excluded due to inability to take medication or illness and did not start the study, eight due to adverse behaviors, noncompliance or insomnia, and were lost to follow-up. Thus, data from a total of 45 children were used for most statistical analyses (Table 1). While five children dropped out after 15 weeks in Phase 2, they were included in the analyses for the length of time they were in the study: two dropped out due to intolerance of the taste/smell, one each due to irritability, recurrent gastrointestinal illness, and for no reason. We measured adherence by asking the caregivers to keep pill diaries, then compared these records to the number of pills that were dispensed and returned. Pill counts were confirmed by the Research Pharmacy and a high degree of compliance was found to be consistent with the cyclocondensation results (Fig. 5). There were no significant differences between the SF and PL groups for children who dropped out after 15 weeks compared to those who completed the study (Additional file 1: Table S2).

**Table 1:** Characteristics of children by intervention group, sulforaphane (SF) or placebo (PL), at baseline

Characteristic	SF (*n* = 22)	PL (*n* = 23)	*p* value^a^
Age (years), mean (SD)	7.4 (3.0)	7.0 (2.5)	0.62
Male sex, *n* (%)	20 (90.9)	20 (90.0)	0.67
Race, *n* (%)			0.37
White	17 (77.3)	15 (65.2)	
Other^b^	5 (22.7)	8 (34.8)	
BMI (kg/m^2^), mean (SD)	16.6 (3.1)	17.4 (3.6)	0.44
Fever responder, *n* (%)	11 (50.0)	8 (34.8)	0.30
Regression, *n* (%)	10 (45.5)	6 (26.1)	0.18
Concomitant medications or therapy, *n* (%)	2 (9.1)	5 (21.7)	0.24
ADOS-2 Calibrated Severity Score, mean (SD)	7.9 (1.4)	7.4 (1.4)	0.31
ADOS-2 social affect score, mean (SD)	13.7 (3.8)	11.8 (3.7)	0.14
ADOS-2 repetitive behaviors score, mean (SD)	4.7 (1.6)	5.7 (1.5)	0.06
Baseline SRS-2 total raw score, mean (SD)	118.2 (26.7)	115.3 (16.6)	0.66
Baseline ABC total score, mean (SD)	74.2 (30.5)	59.7 (23.6)	0.08
Baseline OACIS-S general level of ASD symptoms/behaviors			0.26
Mild/moderate, *n* (%)	7 (31.8)	7 (30.4)	
Marked, *n* (%)	7 (31.8)	12 (52.2)	
Severe, *n* (%)	8 (36.4)	4 (17.4)	
Baseline overall Vineland score^c^	57.5 (11.9)	57.6 (6.7)	0.98
Baseline Leiter composite IQ Score^c^	70.4 (30.8)	70.8 (22.1)	0.96

*BMI* body mass index, *SRS-2* social responsiveness scale 2, *ABC* Aberrant Behavior Checklist, *OACIS-S* Ohio Autism Clinical Impressions Scale (or clinical global impression)—severity, *ASD* autism spectrum disorders, *IQ* intelligence quotient

^a^Chi-square for binary or categorical variables; *t* test for continuous variables

^b^Other race includes Asian, mixed or unknown

^c^*N* = 18 for SF group, *N* = 22 for PL group

Side effects of SF treatment included insomnia, irritability and intolerance of the taste and smell. One child was found to have Hashimoto’s thyroiditis with elevated thyroid stimulating hormone (TSH); when unblinded, he was found to be taking PL. A pediatric endocrinologist determined that this was unrelated to the clinical trial, the child was appropriately treated and then continued to Phase 2. Another child (who had been on PL during Phase 1) took SF for 2 weeks in Phase 2, then discontinued due to taste of SF and 1 month later (without treatment) had new onset of seizures. In both children, the DSMB required that they be unblinded early and concluded that there was no relationship to treatment with SF. Blinding continued for all other children until the end of the study.

Clinical laboratory studies (complete blood count, serum chemistry profile, TSH and urinalysis) at all other visits were within normal limits and no serious side effects were attributed to the treatment (data not shown).

### Ohio Autism Clinical Impressions Scale (OACIS)—Improvement

In this primary outcome measure, most ratings on the OACIS-I were in the 3–4 range (minimally improved or no change), with occasional ratings of 2 (much improved) and rarely 5 (minimally worse). Despite efforts to provide a calm atmosphere, children regularly showed increased anxiety in the clinical setting and especially with phlebotomy at each visit.

Looking at changes from baseline, no statistically significant changes were found between treatment and PL groups on the OACIS-I in the general level of autism (total score) at 7 and 15 weeks (Table 2). The OACIS-I total score (and all subscale scores) improved in both groups when on SF, and while not significant, the effect sizes for the total score at weeks 7 and 15 were small but positive, suggesting a possible trend toward greater improvement in those on treatment with SF. At week 22, when both groups were exposed during the open-label phase, the effect was neutralized, and by week 30 the estimate switched direction in favor of the (former) PL group, though small in magnitude. Both groups displayed washout effects at week 36. Across some subscales, including social interaction severity, aberrant abnormal behaviors and nonverbal communication, the SF group showed gradual improvement while the PL group demonstrated a quick modest improvement at 7 weeks that plateaued at 15 weeks (Table 2). At 22 weeks when all participants were exposed to SF, the (former) PL group showed greater improvement than the SF group (Social Interaction Severity, paired *t* test *p* = 0.01). This effect was also observed in the effect size estimates, specifically for social interaction severity, where the magnitude of effect drastically increased at week 22. This was the only significant effect size across all OACIS-I subscales.

**Table 2:** OACIS-I scores mean unit change from baseline (week 0) at each visit by intervention group, sulforaphane (SF) and placebo (PL), and standardized mean difference of SF compared to PL on OACIS-I scores

OACIS-I subscale	Paired *t* test^a^					Effect size^b^
	SF group *N*	SF group Mean (SD)	PL group *N*	PL group Mean (SD)	*p* value	Cohen’s *d* (95% CI)
Total OACIS-I						
Week 7	17	0.29 (0.69)	17	0.18 (0.39)	0.543	0.21 (− 0.46, 0.88)
Week 15	17	0.29 (0.59)	17	0.24 (0.56)	0.750	0.10 (− 0.52, 0.72)
Week 22	16	0.50 (0.73)	16	0.50 (0.63)	1.000	0.00 (− 0.69, 0.69)
Week 30	15	0.50 (0.68)	15	0.73 (0.78)	0.404	− 0.14 (− 0.90, 0.61)
Week 36	13	0.31 (0.75)	13	0.15 (0.38)	0.502	0.26 (− 0.49, 0.99)
Social interaction severity						
Week 7	17	0.35 (0.70)	17	0.47 (0.62)	0.652	− 0.18 (− 0.93, 0.58)
Week 15	17	0.41 (0.71)	17	0.47 (0.51)	0.773	− 0.09 (− 0.73, 0.54)
Week 22	16	0.63 (0.81)	16	1.31 (0.60)	**0.007**	− **0.97 (**− **1.65, **− **0.26)**
Week 30	15	1.00 (0.83)	15	1.20 (0.61)	0.384	− 0.28 (− 0.89, 0.34)
Week 36	13	0.35 (0.63)	13	0.50 (0.54)	0.337	− 0.26 (− 0.78, 0.27)
Aberrant abnormal behaviors						
Week 7	17	0.06 (0.43)	17	0.24 (0.56)	0.422	− 0.35 (− 1.20, 0.50)
Week 15	17	0.24 (0.44)	17	0.29 (0.69)	0.773	− 0.10 (− 0.78, 0.58)
Week 22	16	0.63 (0.81)	16	0.50 (0.89)	0.718	0.15 (− 0.64, 0.93)
Week 30	15	0.67 (0.90)	15	0.57 (0.86)	0.777	0.11 (− 0.66, 0.88)
Week 36	13	0.15 (0.80)	13	0.08 (0.49)	0.794	0.12 (− 0.74, 0.96)
Repetitive behaviors						
Week 7	17	0.12 (0.33)	17	0.12 (0.33)	1.000	0.00 (− 0.72, 0.72)
Week 15	17	0.06 (0.43)	17	0.12 (0.49)	0.750	− 0.13 (− 0.90, 0.65)
Week 22	16	0.50 (0.63)	16	0.56 (0.89)	0.823	− 0.08 (− 0.79, 0.63)
Week 30	15	0.40 (0.63)	15	0.53 (0.85)	0.658	− 0.18 (− 0.94, 0.60)
Week 36	13	0.15 (0.55)	13	0.15 (0.55)	1.000	0.00 (− 0.80, 0.80)
Verbal communication						
Week 7	17	0.24 (0.56)	17	0.59 (0.62)	0.138	− 0.60 (− 1.37, 0.19)
Week 15	17	0.41 (0.71)	17	0.79 (0.77)	0.109	− 0.51 (− 1.13, 0.11)
Week 22	16	0.63 (0.89)	16	1.28 (0.63)	0.062	− 0.85 (− 1.72, 0.04)
Week 30	15	0.80 (0.84)	15	1.17 (0.84)	0.228	− 0.44 (− 1.13, 0.27)
Week 36	13	0.38 (0.65)	13	0.81 (0.75)	0.182	− 0.60 (− 1.46, 0.28)
Nonverbal communication						
Week 7	17	0.18 (0.53)	17	0.29 (0.47)	0.496	− 0.24 (− 0.90, 0.44)
Week 15	17	0.29 (0.69)	17	0.24 (0.44)	0.750	0.10 (− 0.52, 0.72)
Week 22	16	0.38 (0.62)	16	0.44 (0.63)	0.806	− 0.10 (− 0.88, 0.69)
Week 30	15	0.33 (0.62)	15	0.53 (0.64)	0.384	− 0.32 (− 1.02, 0.39)
Week 36	13	0.15 (0.38)	13	0.08 (0.28)	0.337	0.23 (− 0.24, 0.69)
Hyperactivity inattention						
Week 7	17	0.24 (0.44)	17	0.59 (0.71)	0.138	− 0.60 (− 1.37, 0.19)
Week 15	17	0.35 (0.49)	17	0.76 (0.83)	0.130	− 0.60 (− 1.36, 0.17)
Week 22	16	0.69 (0.79)	16	1.00 (0.89)	0.352	− 0.37 (− 1.13, 0.40)
Week 30	15	0.63 (0.77)	15	0.97 (1.08)	0.420	− 0.36 (− 1.20, 0.50)
Week 36	13	0.08 (0.64)	13	0.23 (0.44)	0.549	− 0.28 (− 1.17, 0.62)
Anxiety						
Week 7	17	0.24 (0.66)	17	0.12 (0.60)	0.608	0.19 (− 0.52, 0.88)
Week 15	17	0.18 (0.53)	17	0.24 (0.75)	0.805	− 0.09 (− 0.80, 0.62)
Week 22	16	0.31 (0.79)	16	0.63 (0.71)	0.352	− 0.41 (− 1.26, 0.45)
Week 30	15	0.37 (0.67)	15	0.40 (0.83)	0.916	− 0.04 (− 0.85, 0.77)
Week 36	13	0.23 (0.60)	13	0.04 (0.32)	0.240	0.40 (− 0.26, 1.05)
Sensory sensitivities						
Week 7	17	0.24 (0.56)	17	0.18 (0.39)	0.750	0.12 (− 0.61, 0.85)
Week 15	17	0.18 (0.39)	17	0.24 (0.44)	0.718	− 0.14 (− 0.89, 0.62)
Week 22	16	0.38 (0.62)	16	0.44 (0.63)	0.806	− 0.10 (− 0.88, 0.69)
Week 30	15	0.33 (0.62)	15	0.47 (0.64)	0.634	− 0.21 (− 1.07, 0.65)
Week 36	13	0.15 (0.55)	13	0.15 (0.38)	1.000	0.00 (− 0.94, 0.94)
Restricted or narrow interests						
Week 7	17	0.06 (0.24)	17	0.06 (0.24)	1.000	0.00 (− 0.69, 0.69)
Week 15	17	0.12 (0.49)	17	0.24 (0.56)	0.543	− 0.22 (− 0.93, 0.49)
Week 22	16	0.19 (0.54)	16	0.31 (0.70)	0.608	− 0.20 (− 0.94, 0.55)
Week 30	15	0.20 (0.56)	15	0.20 (0.56)	1.000	0.00 (− 0.76, 0.76)
Week 36	13	− 0.07 (0.28)	13	0.07 (0.28)	0.165	− 0.55 (− 1.31, 0.22)

Bold signifies *p* < 0.05

*OACIS-I* Ohio Autism Clinical Impressions Scale (or clinical global impression)—improvement

^a^Paired *t* test matched for sex and fever response

^b^Standardized mean difference for matched pairs

Since the SF treatment group included more children with severe ASD compared to the PL group, we performed a separate analysis of some OACIS-I scores using paired *t* tests (with limited *N*) and effect sizes excluding these participants. While the patterns were similar compared to results including all children, they were shifted more in the direction of the SF group (Additional file 1: Table S3). The Cohen’s d estimates for the total score, while still not significant, were over twofold (0.21–0.48) and fivefold (0.10–0.55) higher, at 7 and 15 weeks, respectively, compared to the full analysis, indicating a medium effect size. For social interaction severity, the entire direction of effect changed in favor of SF. Further, the effect sizes at week 7 for both verbal communication and hyperactivity inattention shifted from − 0.60 to 0, and similar patterns were observed across visits.

### Social responsiveness scale-2 (SRS-2)

**Fig. 3:**
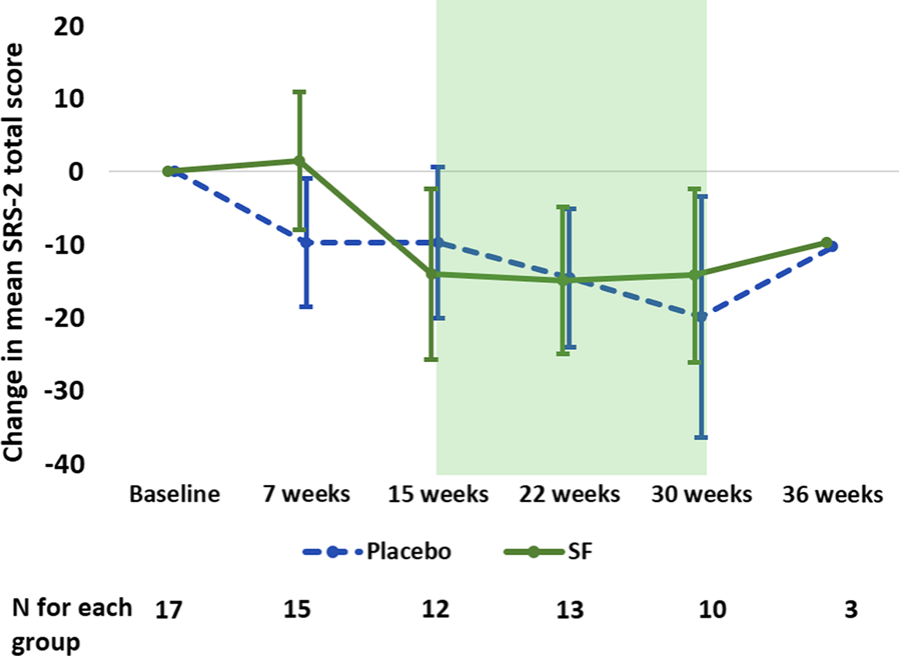
Change in mean (95% CI) total SRS-2 raw scores from baseline using sex- and fever response-matched pairs. Scores between sulforaphane (SF) and placebo (PL) groups did not differ significantly. Both groups’ scores improved during the open label phase after 15 weeks (shaded box). *Note*: 95% CI not shown for 36 weeks due to small sample size; see Additional file 1: Table S5 for mean (SD)

In a paired group-wise comparison of total “raw” scores (unadjusted for general population, since all children had ASD), there were no significant differences between treatment and PL groups at 7 or 15 weeks. Scores trended downward, indicating improvement, when both groups were on SF at 22 weeks (Fig. 3).

The combined exposure group included all children from the SF group at weeks 7 and 15, *and* all children from the placebo group during their first two visits on intervention (weeks 22 and 30 in the trial but considered their first 7 and 15 weeks of exposure to SF). These values were compared to their “baseline” at 15 weeks. Exposure to SF for the PL group was only during the open-label phase (weeks 16–30). A repeated measures analysis of SRS-2 total and subscale scores for length of exposure to SF showed significant improvements compared to baseline (*p* < 0.05 to *p* < 0.001) in most subscales at 15 weeks for the combined SF exposure group (7–15 weeks) and carried over into the intervention (SF) only group (16–30 weeks), compared to baseline (Table 3). There were minimal to no differences between 30 and 36 or 0 and 36 weeks. For additional SRS-2 data, see Additional file 1: Tables S4–6.

**Table 3:** SRS-2 length of sulforaphane (SF) exposure analysis

SRS variable	*N*	Intervention (SF)^a^			PL (non-exposure)	
		F-statistic	*p* value	*N*	F-statistic	*p* value
Total score						
7 weeks	38	1.64	0.209	21	9.95	**0.005**
15 weeks	34	11.64	**< 0.001**	17	5.40	**0.017**
22 weeks	19	6.12	**0.006**			
30 weeks	14	6.17	**0.009**			
Social awareness						
7 weeks	38	1.39	0.247	21	0.13	0.724
15 weeks	34	8.12	**0.001**	17	1.16	0.339
22 weeks	19	6.88	**0.004**			
30 weeks	14	6.46	**0.008**			
Social cognition						
7 weeks	38	0.02	0.879	21	8.40	**0.009**
15 weeks	34	3.26	**0.052**	17	4.58	**0.028**
22 weeks	19	3.31	**0.047**			
30 weeks	14	5.34	**0.015**			
Social communication						
7 weeks	38	1.55	0.221	21	8.20	**0.010**
15 weeks	34	11.03	**< 0.001**	17	3.65	0.051
22 weeks	19	5.54	**0.008**			
30 weeks	14	2.94	0.076			
Social motivation						
7 weeks	38	0.01	0.916	21	3.66	0.070
15 weeks	34	2.81	0.075	17	2.02	0.168
22 weeks	19	2.21	0.126			
30 weeks	14	5.31	**0.015**			
RRB^b^						
7 weeks	38	4.37	**0.044**	21	5.29	**0.032**
15 weeks	34	10.14	**< 0.001**	17	3.33	0.064
22 weeks	19	5.26	**0.010**			
30 weeks	14	3.85	**0.038**			
SCI^c^						
7 weeks	38	0.67	0.417	21	9.56	**0.006**
15 weeks	34	10.78	** < 0.001**	17	4.59	**0.028**
22 weeks	19	6.21	**0.005**			
30 weeks	14	7.38	**0.005**			

Bold signifies *p* < 0.05

MANOVA test for repeated measures used (compared to baseline at 0 weeks)

^a^N for SF group at 7 and 15 weeks includes participants from the initial PL group while they were on SF at 22 and 30 weeks (their first 7 and 15 weeks of exposure)

^b^Restricted interests and repetitive behavior

^c^Social communication and interaction

### Aberrant Behavior Checklist (ABC)

**Fig. 4:**
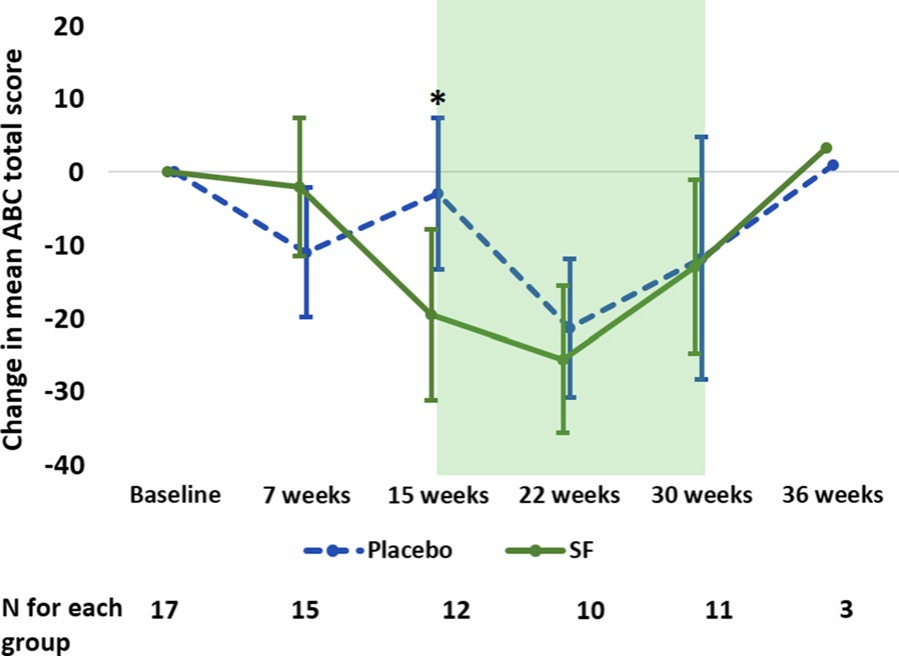
Change in mean (95% CI) total ABC raw scores from baseline using sex- and fever response-matched pairs. Change in mean score was significantly different between the sulforaphane (SF) and placebo (PL) groups at 15 weeks. Both groups’ scores improved during the open label phase after 15 weeks (shaded box). *Note*: 95% CI not shown for week 36 due to small sample size; see Additional file 1: Table S8 for mean (SD). *p = 0.02

A paired group-wise analysis of ABC scores showed no significant difference between treatment and PL groups at 7 weeks; however, at 15 weeks, there was a significantly greater decrease in the total score in the SF group, denoting improvement in the SF group compared to PL (*p* < 0.02; Fig. 4). Cohen’s *d* at 15 weeks was − 0.96 (95% CI − 1.73, − 0.15), a difference of close to one full standard deviation, indicating a large effect (Additional file 1: Table S8). Similar effect sizes were seen for inappropriate speech at weeks 15 (− 0.85, 95% CI − 1.49, − 0.19) and 30 (− 0.99, 95% CI − 1.89, − 0.05). The effect size for hyperactivity and inattention was even greater at both 15 (− 1.67, 95% CI − 2.70, − 0.59) and 22 weeks (− 1.22, 95% CI − 2.18, − 0.22). For stereotypy, the (former) PL group showed a greater decrease at 22 weeks when all subjects were on SF treatment (1.54, 95% CI 0.49, 2.55). For additional ABC data, see Additional file 1: Tables S7–9 in the Supplementary Information.

Repeated measures analysis of ABC total score and subscale scores for length of exposure to SF showed significant improvements (*p* < 0.05 to *p* < 0.001) compared to baseline values for the combined SF exposure group (7–15 weeks) and carried over to 22 weeks for total score and hyperactivity in the SF-only group (Table 4). Decreased scores (showing improvement) were evident by 7 weeks of treatment and were more marked in the SF compared to the PL group. There were again minimal to no differences between 30 and 36 weeks or between 0 and 36 weeks (Additional file 1: Tables S7–9).

**Table 4:** ABC length of sulforaphane (SF) exposure analysis

ABC variable	Intervention (SF)^a^			Non-exposure (PL)		
	*N*	F-statistic	*p* value	*N*	F-statistic	*p* value
Total score						
7 weeks	38	9.59	**0.004**	21	4.76	**0.041**
15 weeks	34	12.74	**0.001**	18	2.02	0.166
22 weeks	16	5.05	**0.016**			
30 weeks	12	2.22	0.156			
Sqrt lethargy^b^						
7 weeks	38	1.55	0.221	21	2.94	0.102
15 weeks	34	4.75	**0.016**	18	1.68	0.218
22 weeks	16	4.20	**0.028**			
30 weeks	12	2.06	0.179			
Sqrt irritability						
7 weeks	38	6.06	**0.019**	21	2.27	0.147
15 weeks	34	9.27	**0.001**	18	0.87	0.436
22 weeks	16	4.03	**0.032**			
30 weeks	12	2.06	0.179			
Sqrt stereotypy						
7 weeks	38	4.85	**0.034**	21	1.74	0.202
15 weeks	34	6.06	**0.006**	18	1.72	0.210
22 weeks	16	1.22	0.343			
30 weeks	12	0.75	0.588			
Hyperactivity						
7 weeks	38	9.62	**0.004**	21	10.15	**0.005**
15 weeks	34	9.95	**< 0.001**	18	4.68	**0.025**
22 weeks	16	5.36	**0.013**			
30 weeks	12	3.26	0.073			
Inappropriate speech						
7 weeks	38	7.85	**0.008**	21	0.27	0.607
15 weeks	33	4.11	**0.026**	18	0.11	0.894
22 weeks	16	3.02	0.068			
30 weeks	12	1.95	0.196			

Bold signifies *p* < 0.05

MANOVA test for repeated measures

^a^*N* for SF group at 7 and 15 weeks includes participants from the initial PL group while they were on SF at 22 and 30 weeks (their first 7 and 15 weeks of exposure)

^b^Sqrt: Square root transformations were conducted on these biomarkers to achieve a more normal distribution

### Fever response and developmental regression

Based on our hypothesis that the “fever effect” in ASD (in which children rapidly and transiently improve in social responsiveness during febrile illnesses) may be related to stimulation of heat shock responses or related mechanisms, and since certain HSPs are up-regulated by SF, we posited that children with the fever effect, as reported by their caregivers, may respond differentially to SF. Our analysis in this study of length of exposure on SRS showed greater responses for *non*-fever responders in total SRS scores, social awareness and social communication at 15 weeks of treatment, compared to fever responders (Additional file 1: Table S10). While this pattern was also observed in the ABC, differences between the two groups were more subtle (Additional file 1: Table S11).

Considering the possible underlying metabolic differences in children with ASD who have a history of developmental regression, we examined length of exposure and found that children in both groups, with and without regression, improved on both the SRS-2 and ABC, especially over the first 15 weeks; however, those without regression improved to a greater degree (Additional file 1: Tables S12–13). In analyses of children with the fever response and developmental regression, the number of children (*N*) was greater in the non-fever responders and in those without developmental regression. Additional results for biomarker analyses with respect to the fever response and developmental regression can be found in the biomarkers section.

### Mixed effects models

For the SRS-2 total score, there was no significant treatment effect overall (across all visits) (*p* = 0.22). However, with treatment held constant, there was a continual improvement in the total score with time (visit), and, after week 7, these changes were significant (*β* for week 30 = − 16.1, *p* = 0.01). All SRS-2 subscales were similar for improvement by visit, regardless of treatment group. Treatment did not have an effect for any of the subscales, except for social awareness, where it was the PL group that improved. However, this improvement was only significant at week 22, after the placebo group had started on SF (*p* = 0.04; Additional file 1: Table S14).

For the ABC, the treatment effect was significant for total score where the PL group improved more than the SF group (*p* < 0.01). However, there was a trend of improvement regardless of treatment group across the 30 weeks, similar to the SRS-2 results. The ABC total score was accompanied by the subscales of lethargy, irritability, hyperactivity and inappropriate speech showing comparable trends with similar improvement over time, holding treatment group constant. There was a reversal in the trend from 22 to 30 weeks, though there was still significant improvement overall (Additional file 1: Table S15). This may have been due to treatment fatigue, in which caregivers’ perception of improvements waned due to the long duration of the study.

Mixed effects modeling was also performed for SF treatment on the ABC and SRS-2 in a sample excluding children with severe ASD. These results revealed no statistically significant beta coefficients for SF compared to Placebo, controlling for number of weeks (data not shown). This was true for the total scores and all subscales (except the irritability subscale in the ABC; *p* = 0.04). However, the coefficient for the SRS-2 total score was 0.55 compared to 4.21 in the full analysis as the coefficients for each of the subscales were lower across the board. Coefficients for each week, controlling for treatment, were similar to results including all children for both the ABC and SRS-2.

### Cyclocondensation data (indicating compliance and bioavailability)

**Fig. 5:**
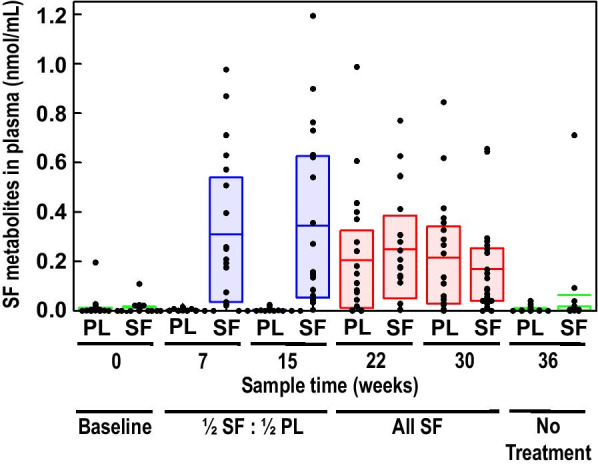
Plasma cyclocondensation of SF metabolite (DTC) levels. “*PL*” directly under the X-axis indicates Placebo arm and “*SF*” indicates Sulforaphane arm. Further annotation indicates actual treatment delivery at times indicated

Plasma levels of SF metabolites, measured by cyclocondensation, showed considerable variability due to variable timing of phlebotomy relative to administration of the SF (from 3 to 8 h) as well as individual variation in metabolism (Table 5 and Fig. 5), similar to observations by Egner et al. [25]. High values of SF for 3 children were omitted from the statistical analysis—2 at baseline and 1 at 36 weeks—because we learned their parents had been giving them supplements on their own. Note that no SF was detected in the PL group at 7 and 15 weeks. There was no statistically significant difference between the two groups when both were taking SF at 22 and 30 weeks, and both returned to baseline by 36 weeks (after 6 weeks without SF). We found no significant association between levels of metabolites and clinical measures of ASD.

**Table 5:** Plasma cyclocondensation means by sulforaphane (SF) and placebo group for each visit

	SF group *N*	SF group nmol DTC/mL^a^ Mean (SD)	PL group *N*	PL group nmol DTC/mL^a^ Mean (SD)	*p* value^b^
Week 0	21	0.007 (0.008)	24	0.006 (0.008)	0.614
Week 7	21	0.299 (0.297)	22	0.003 (0.005)	**< 0.0001**
Week 15	21	0.329 (0.350)	24	0.005 (0.008)	**< 0.0001**
Week 22	19	0.248 (0.232)	20	0.205 (0.253)	0.582
Week 30	22	0.165 (0.183)	20	0.214 (0.228)	0.451
Week 36	15	0.015 (0.024)	16	0.008 (0.012)	0.286

Bold signifies *p* < 0.05

^a^DTC: dithiocarbamates, SF metabolites in plasma detected by cyclocondensation

^b^*p* values based on *t* test

### Markers of glutathione redox status

The data were analyzed with and without hemolyzed samples. Because there was no significant difference when analyses included or excluded hemolyzed samples, the data in Tables 6 and 7 reflect analyses with all samples included. Compared to baseline values, free reduced (fGSH), oxidized (fGSSG) and total glutathione (tGSH) did not differ within or between the SF and placebo groups. Oxidative stress is best defined as an imbalance between antioxidant and pro-oxidant potential and can be quantified by the ratio of GSH/GSSG (reduced to oxidized glutathione) [33]. The ratio of fGSH/fGSSG and tGSH/fGSSG differed between groups at 15 weeks (*p* = 0.002, 0.03, respectively; Table 6, Additional file 1: Table S16). Treatment with SF was associated with lower ratios of both fGSH/fGSSG and tGSH/fGSSG. When examined in terms of SF exposure alone (for both SF and the PL group when taking SF), similar decreases in fGSH/fGSSG and tGSH/fGSSG were present at 15 and 30 weeks (Table 7, Additional file 1: Table S17).

**Table 6:** Effect of treatment at first 15 weeks comparing sulforaphane (SF) and placebo (PL) groups

Biomarker	SF (exposure)			PL (non-exposure)		
	*N*	F-statistic	*p* value	*N*	F-statistic	*p* value
Free reduced GSH^a^	22	1.51	0.232	23	0.11	0.746
Total GSH^a^	22	0.00	0.945	23	0.08	0.776
GSSG^a^	22	1.97	0.175	23	0.46	0.505
Free GSH:GSSG	22	12.72	**0.002**	23	0.87	0.361
Total GSH:GSSG	22	5.16	**0.034**	23	0.03	0.875

Bold signifies *p* < 0.05

MANOVA test used for repeated measures (compared to baseline at 0 weeks)

^a^Natural log transformed

**Table 7:** Effect of treatment exposure for combined sulforaphane (SF) and placebo (PL) groups at 15 weeks and for SF-only group at 30 weeks

Biomarker	Intervention (exposure)			Change
	*N*	F-statistic	*p* value	
Free reduced GSH^a^				
15 weeks	40	1.95	0.170	–
30 weeks	22	0.75	0.485	
Total GSH^a^				
15 weeks	40	0.11	0.746	–
30 weeks	22	0.06	0.946	
GSSG^a^				
15 weeks	40	0.15	0.701	↑
30 weeks	22	2.32	0.124	
Free GSH:GSSG				
15 weeks	40	3.90	0.055	↓
30 weeks	22	8.12	**0.003**	
Total GSH:GSSG				
15 weeks	40	1.32	0.258	↓
30 weeks	22	3.60	**0.046**	

Bold signifies *p* < 0.05

MANOVA test for repeated measures (compared to baseline at 0 weeks); analysis includes participants from the initial PL group while they were on SF at 22 and 30 weeks (their first 7 and 15 weeks of exposure)

^a^Natural log transformed

### Cytoprotective markers

**Fig. 6:**
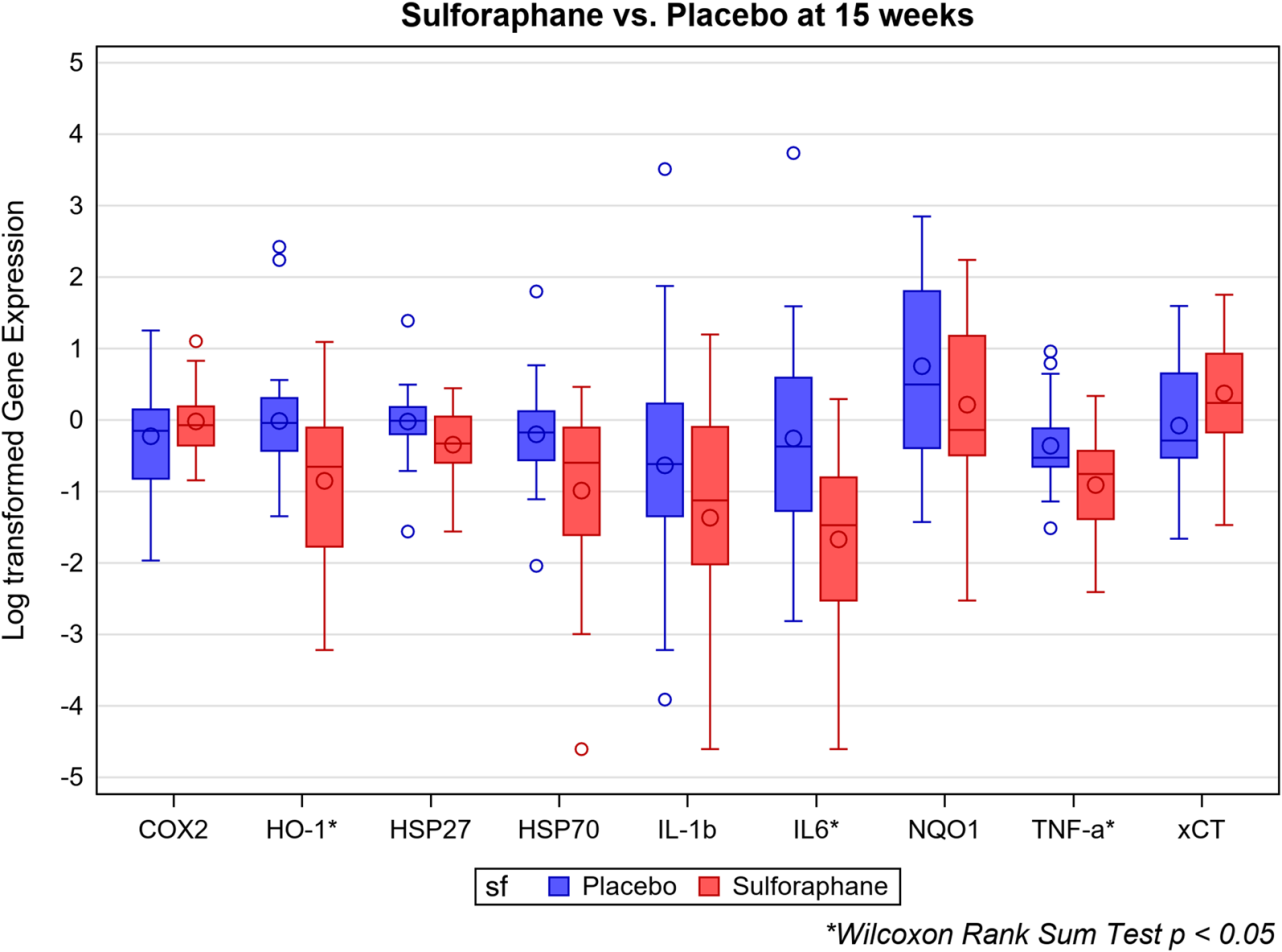
Natural log of relative gene expression for biomarkers, SF versus PL, from baseline to 15 weeks. *N* = 42. Small circles outside of the boxes denote outliers. Large circles inside the boxes denote means; center horizontal lines inside the boxes denote medians

**Fig. 7:**
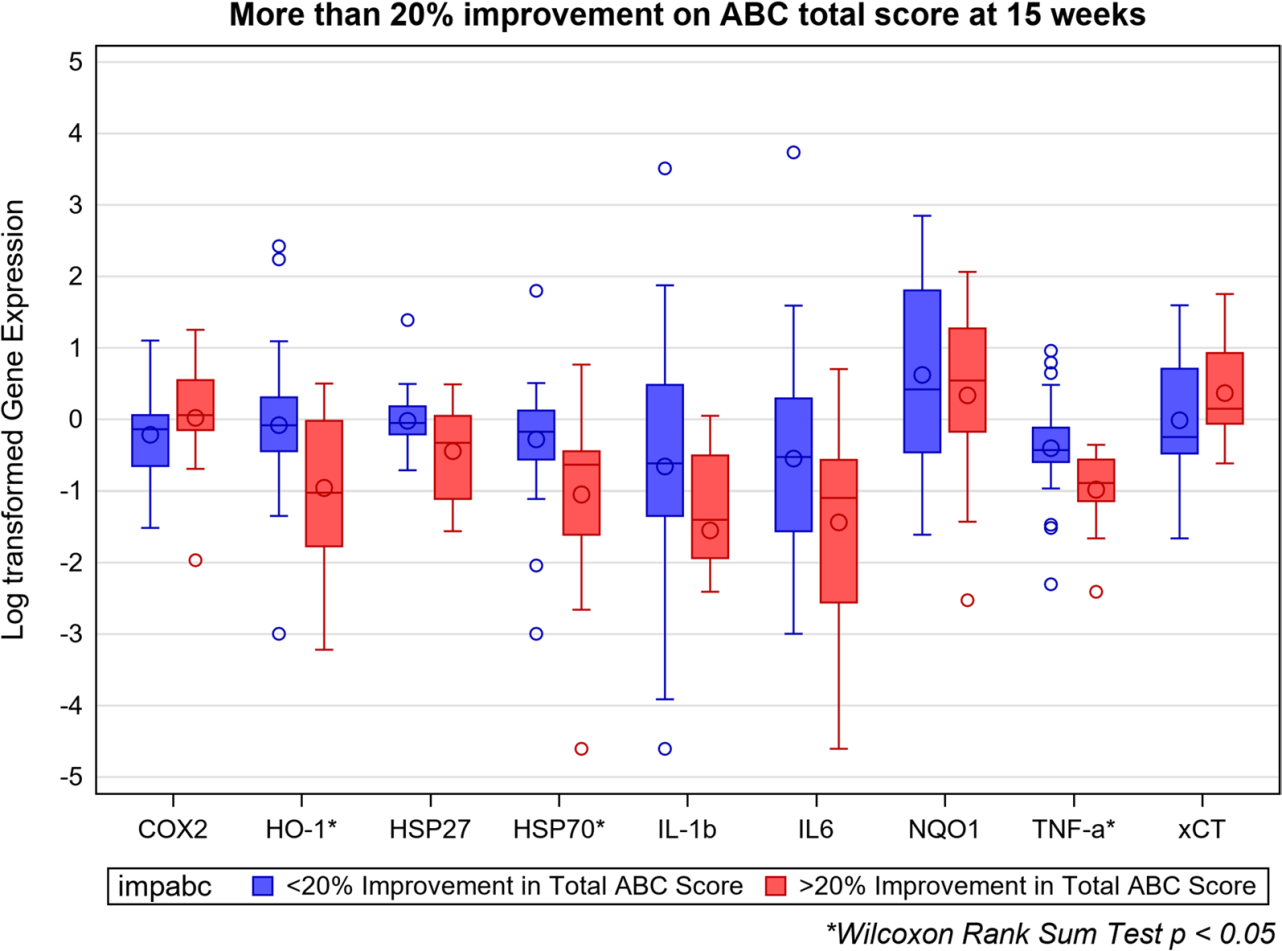
Natural log of relative gene expression of biomarkers for participants with more than 20% improvement on ABC total scores from baseline to 15 weeks. *N* = 42. Small circles outside of the boxes denote outliers. Large circles inside the boxes denote means; center horizontal lines inside the boxes denote medians

After 15 weeks of treatment with SF, the (natural log) change from baseline in gene expression of HO-1 was significantly lower than those on PL (*p* = 0.01; Table 7). Values for NQO1 were lower (N.S.), and xCT did not differ between participants taking SF and PL. Using the Wilcoxon rank sum test, HO-1 expression was lower in those taking SF at 15 weeks, and in children whose total ABC scores decreased by more than 20% compared to their baseline (*p* < 0.05), whereas NQO1 and xCT did not differ significantly (Figs. 6, 7).

### Heat shock proteins

Gene expression for HSP70 at 15 weeks was significantly lower in children taking SF compared to PL (*p* = 0.03), and there was a trend toward lower HSP27 (*p* = 0.08; Table 8). HSP70 was also lower in children taking SF whose total ABC scores decreased more than 20% (*p* < 0.05; Fig. 6). HSP27, HSP70 and HO-1 all returned to baseline at 36 weeks. The combination of decreased HSP27 and HSP70 differed from baseline for treatment with SF (*p* = 0.06) but not PL (*p* = 0.3).

**Table 8:** Univariate regression (*β*) coefficients for sulforaphane compared to placebo for biomarker gene expression (change from baseline)

	*β* coefficient	95% CI	*p* value
HO-1			
Week 7	− 0.50	(− 1.13, 0.14)	0.121
Week 15	− 0.84	(− 1.50, − 0.19)	**0.013**
HSP70			
Week 7	− 0.41	(− 0.90, 0.07)	0.091
Week 15	− 0.78	(− 1.45, − 0.10)	**0.025**
HSP27			
Week 7	− 0.04	(− 0.27, 0.18)	0.693
Week 15	− 0.31	(− 0.66, 0.04)	0.078
IL-6			
Week 7	− 0.39	(− 0.94, 0.17)	0.166
Week 15	− 1.31	(− 2.22, − 0.39)	**0.006**
IL-1β			
Week 7	− 1.13	(− 2.11, − 0.14)	**0.026**
Week 15	− 0.76	(− 1.88, 0.35)	0.174
Week 22	− 0.26	(− 1.36, 0.83)	0.627
Week 30	− 1.32	(− 2.55, − 0.09)	**0.037**
TNF-α			
Week 7	− 0.18	(− 0.62, 0.25)	0.394
Week 15	− 0.56	(− 0.98, − 0.14)	**0.010**

Bold signifies *p* < 0.05

Analysis based on natural log transformed fold change values. See complete Additional file 1: Table S18

### Inflammatory markers

In children taking SF for 15 weeks, IL-6 and TNF-α gene expression was significantly lower (*p* = 0.006, 0.01) compared to those taking PL. The changes in levels of IL-1β expression from baseline to weeks 7 and 30 were significantly greater in participants on SF than those on PL (*p* = 0.03, 0.04) but not from baseline to 15 or 22 weeks (Table 8). Accordingly, Wilcoxon rank sum scores were significantly decreased at 15 weeks for IL-6 and TNF-α for children taking SF, and for TNF-α in those whose total ABC scores decreased by > 20% (Figs. 6, 7). The combination of decreased TNF-α and IL-6 differed for treatment with SF compared to baseline at 15 weeks (*p* < 0.05) but not PL (*p* = 0.6).

### Biomarkers of fever response and developmental regression

For participants with a history of the fever effect compared to those without, there was significantly greater gene expression for xCT, HSP70 and COX-2 at baseline (*p* = 0.004, 0.006 and 0.001, respectively; data not shown). After 15 weeks of treatment with SF for fever responders in both groups (0–15 weeks for the SF group and 16–30 weeks for the Placebo group), there was reduced expression of xCT (*p* = 0.03) and greater expression of HSP70 (*p* = 0.04), COX-2 (*p* = 0.02) and TNF-α (*p* = 0.04; Table 9). In participants with a history of developmental regression, there were no statistically significant differences in gene expression at baseline or after 15 weeks of treatment with SF, compared to their baseline values (Additional file 1: Table S19).

**Table 9:** Mean biomarker gene expression at 15 weeks of exposure to sulforaphane by presence or absence of fever effect (natural log transformed values for change from baseline)

	Fever effect *N*	Fever effect Mean (95% CI)	No fever effect *N*	No fever effect Mean (95% CI)	*p* value^†^
NQO1	16	0.33 (− 0.34, 1.00)	20	0.93 (0.26, 1.60)	0.196
xCT	17	− 0.24 (− 0.72, 0.23)	21	0.51 (0.02, 0.99)	**0.027**
HO-1	17	− 0.45 (− 1.08, 0.18)	21	− 0.40 (− 1.07, 0.28)	0.906
HSP70	17	0.02 (− 0.22, 0.26)	21	− 0.35 (− 0.60, − 0.09)	**0.037**
HSP27	17	− 0.52 (− 1.12, 0.08)	21	− 0.70 (− 1.42, 0.02)	0.697
IL-6	17	− 1.59 (− 2.21, − 0.98)	20	− 0.98 (− 1.69, − 0.27)	0.187
IL-1β	16	− 0.61 (− 1.27, 0.06)	20	− 1.12 (− 2.22, − 0.02)	0.434
COX-2	17	0.08 (− 0.26, 0.41)	21	− 0.36 (− 0.55, − 0.18)	**0.015**
TNF-α	17	− 0.30 (− 0.68, 0.07)	21	− 0.89 (− 1.32, − 0.46)	**0.043**

Bold signifies *p* < 0.05

*p* value based on *t* test

### Mitochondrial function

**Fig. 8:**
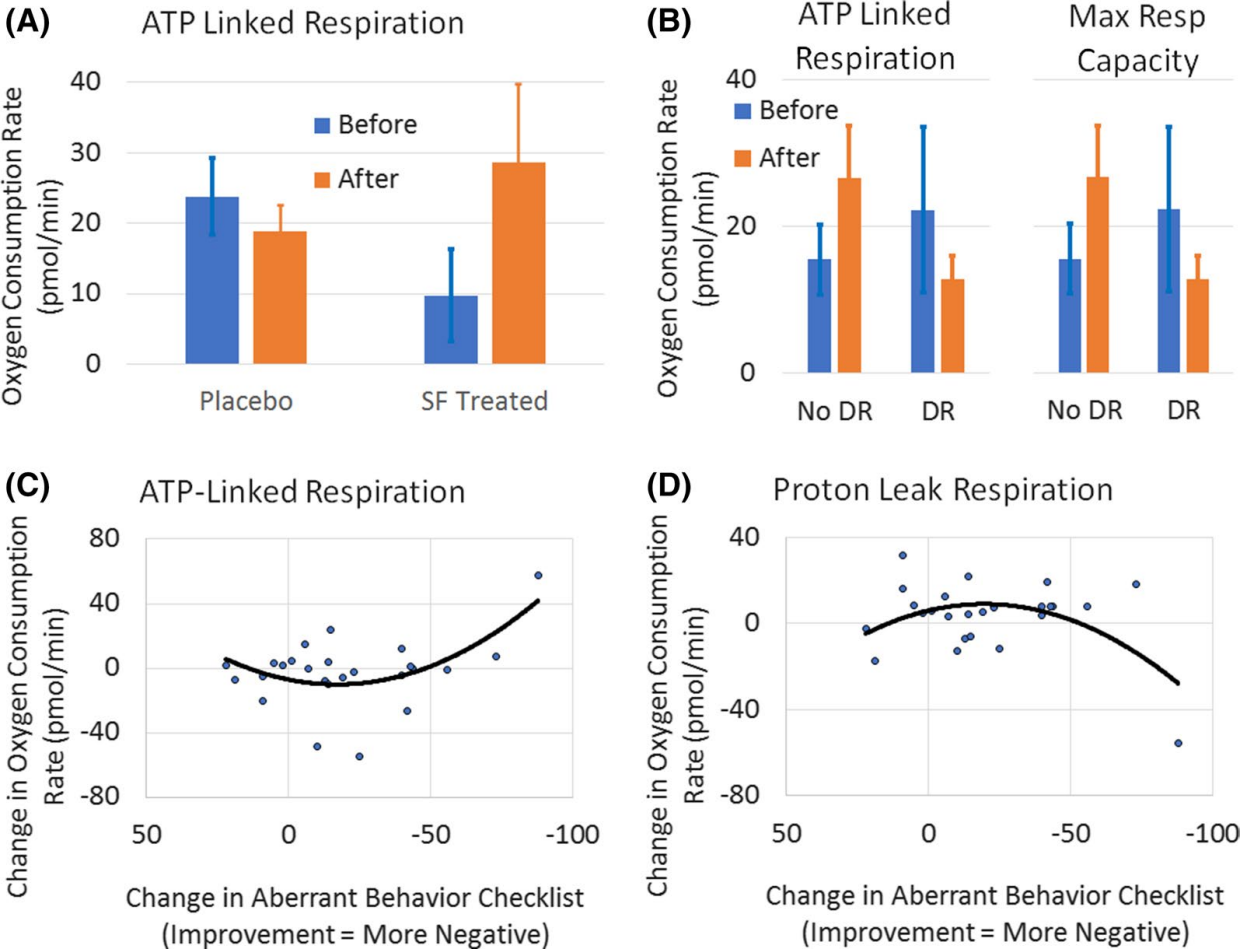
Relation between respiratory parameters and both treatment with Sulforaphane (SF) and change in Aberrant Behavior Checklist (ABC) scores. **a** ATP-Linked respiration significantly increased (*p* < 0.05) with SF treatment (*N* = 13) as compared to PL (*N* = 14); **b** Individuals with developmental regression demonstrated a trend for decreased ATP-Linked Respiration and Maximal Respiratory Capacity over the DBPC treatment period while those without developmental regression demonstrated the opposite trend. **c** Greater improvement in ABC scores was associated with a greater increase in ATP-Linked Respiration and **d** decrease in Proton Leak Respiration, across the entire study period

Twenty-seven participants (13 treated with SF, 14 with PL; 21 without and 6 with developmental regression) had valid Seahorse data for analysis. For the placebo-controlled (Phase 1) of the study ATP-Linked Respiration increased for the individuals treated with SF but not for those treated with PL [*F*(1,24) = 4.41, *p* < 0.05] and there was no significant difference between the groups at baseline (Fig. 8a). There was a trend for the individuals with developmental regression to demonstrate a decrease in ATP-Linked Respiration [*F*(1,24) = 2.37, *p* = 0.14] and Maximal Reserve Capacity [*F*(1,24) = 2.88, *p* = 0.10] over the treatment period (Fig. 8b). There was no significant overall systematic change in respiratory parameters across the open-label phase of the study.

When examining the entire treatment period from baseline to the end of the open label phase, the change in the ABC scores was nonlinearly significantly related to change in ATP-Linked Respiration [Linear *F*(2,40) = 6.39, *p* < 0.01; Quadratic *F*(2,40) = 14.21, *p* < 0.001; *r* = 0.55, *p* < 0.01] (Fig. 8c) and Proton-Leak Respiration [Linear *F*(2,40) = 3.42, *p* < 0.05; Quadratic *F*(2,40) = 11.29, *p* < 0.001; *r* = 0.50; *p* < 0.01] (Fig. 8d).

## Discussion

Sulforaphane has meaningful potential for treating children with ASD due to its many known mechanistic effects, clinical benefits and lack of toxicity [5, 34]. Small but non-statistically significant effects of SF treatment were observed on the general level of autism and subscales using the OACIS-I, our primary outcome measure, at weeks 7 and 15. Greater effect sizes were observed among a subsample of children with non-severe ASD. These effects equilibrated when both SF and PL groups were exposed to treatment at 22 and 30 weeks, and both groups displayed washout effects. Notably, the social interaction severity subscale improved greatly among the (former) PL group at week 22 when taking SF. While it may have been an “open-label” effect, this finding coincides with the results from our previous trial of SF in young men [6]. There was also significant improvement measured by the ABC but not the SRS-2. A non-randomized analysis for length of exposure to SF showed significant improvements on both the ABC and SRS-2. Decreased total scores (showing improvement) on the SRS-2 and ABC ranged from 13 to 31%, respectively, and changes of greater magnitude took place among subscales signaling improvements in response to SF.

This was a long clinical trial (conducted over 3 years) with each research subject required to participate for 36 weeks, with 6 outpatient visits that induced considerable anxiety for the children, especially due to phlebotomies for clinical laboratory safety and biomarker studies at each visit when clinical assessments were done. We confirmed that children received the SF, as shown by cyclocondensation measurements, and there were no signs of toxicity on clinical laboratory tests or serious adverse events. Both the bioavailability of SF as judged by cyclocondensation values and the spread of those values were remarkably like those previously reported in studies of adults, dosed with a similar quantity of a SF-rich preparation (150 μmoles SF or about 2.2 μmoles/kg body weight) [35, 36]. This similarity in bioavailability as well as an alignment in bioavailability with other shorter-term interventions utilizing the same or similar dosing modalities [26, 27] also validates our decision not to limit intake of cruciferous vegetables or other foods. Caregivers were only asked not to administer SF with a heavy meal, to give it at about the same time each day and to maintain a list of concurrent medications and supplements. It was highly unlikely that any of the subjects would be consuming large amounts of cruciferous vegetables and consumption of SF supplements would have been apparent in the cyclocondensation data during the placebo phase. Food frequency questionnaires would have been of extremely limited value in this cohort and would have contributed even further to treatment fatigue.

There was considerable treatment fatigue over time, as shown by increases in parents’ SRS-2 and ABC scores at 22 and 30 weeks for most subscales, when all were taking SF and scores trended back toward the baseline, irrespective of the subjects’ length of exposure to SF. Improvements were also noted on the ABC and SRS-2 by caregivers in the absence of SF. These factors, along with known placebo effects in clinical trials in ASD, contributed to difficulties in estimating clinical changes [37, 38]. Children with ASD also show considerable inherent variability over time in their behavior, sleep and ability to communicate that makes clinical estimates of ASD symptoms problematic at any given time [39]. These factors confound clinical trials in ASD and might be approached in future trials using physiological measurements such as eye tracking and actigraphy [40, 41].

The source of SF in this trial was GR with added myrosinase, the combination of which has been shown to provide adequate substrate for SF in small trials in typical adults [25–27], although its conversion to SF was less than, and more varied than a SF-rich preparation. In our previous clinical trial using a SF-rich preparation in young men [6], clinical improvements were more obvious and constant over time to clinicians as well as caregivers. It is also possible that children may metabolize SF differently than young men. In our pilot study for this trial [5], we noted variability in turnover of GR to SF, with average urinary conversion like the previous study in young adults. In the main study, there were notable exceptional clinical responses in several children, as reported by their caregivers that were not consistently evident during our clinical assessments prior to the phlebotomies. Clinical responses to GR + myrosinase (as the source of SF) were more evident to parents, whose ratings on the ABC and SRS showed significant improvements, despite considerable placebo effects.

The clinical efficacy of SF in ASD in children in this trial and our previous trial in young men [6] compares favorably to intranasal oxytocin [42] and bumetanide [43] in recent placebo-controlled clinical trials. Although outcome measures and statistical power differed between this study and the other two, SF appears to enhance socialization like oxytocin and improves other core features of ASD similar to bumetanide. As a “natural” dietary component, SF may have less potential for toxicity than either drug with long term use, although further studies of SF are needed in children with ASD, for both long-term safety and efficacy.

Biomarkers provided strong evidence for the biologic effects of SF, especially with respect to markers of glutathione redox status, inflammatory cytokines and mitochondrial function. Decreases in the plasma redox ratio of free and total glutathione were present at 15 and 30 weeks on treatment, primarily due to increases in oxidized GSSG. These results are consistent with SF-induced increased turnover of anti-inflammatory GSH as well as cellular export of GSSG in an attempt to control intracellular oxidative stress [44]. HO-1 functions to couple activation of mitochondrial biogenesis to anti-inflammatory cytokine expression [45]; it was initially increased in the pilot study, then paradoxically decreased in the main study, on continued treatment for longer periods with SF. Increased HO-1 is consistent with decreases in the proinflammatory cytokines we observed initially in IL-6, IL-1β and TNF-α. Decreased levels of the cytokines continued after HO-1 returned to baseline with longer duration of treatment and suggest a decreased inflammatory state. These cytokines, as measured in the serum, are usually elevated in children with ASD [46–48], but were decreased on treatment with SF: IL-6 and TNF-α at 15 (but not 30) weeks. The most consistent change was in TNF-α, increases in which have been frequently associated with ASD, both in serum and cerebrospinal fluid [47–49].

The findings for heat shock proteins, HO-1 and cytokines suggest dynamic changes over time in the children’s metabolism, possibly inherent in ASD or due to SF. Heat shock proteins are expected to *increase* with fever and in response to SF [17], which we observed in our pilot study (along with HO-1 and NQO1) after just 2 weeks of treatment [5]. Cytokines (IL-6, IL-1β and TNF-α) were all decreased at 2 weeks, which persisted later in the main study, but returned toward baseline after 15 weeks of treatment. We speculate that SF may induce changes over time that modify regulation in several systems at the cellular level. For example, SF may initially increase expression of heat shock proteins and HO-1 that later decrease and return to a new equilibrium state with continued treatment. Similar changes may occur in cytokines, whereas decreased markers of oxidative stress may persist. These and other effects of SF may become apparent in ongoing studies of urine metabolomics from this trial [50].

Mitochondrial function improved in the SF group as compared to the placebo group during the placebo-controlled phase of the study and improvement in mitochondrial parameters correlated with improvements on the ABC scale. This suggests that the clinical response to SF was associated with changes in mitochondrial function and that large intrasubject variability in this study was linked to underlying biological responses. The increase in ATP-Linked Respiration associated with improvement in ABC scores suggests that those individuals who showed improvements in behavior also had improved mitochondrial capacity to produce ATP. Individuals who showed an improvement in ABC scores also showed a decrease in Proton Leak Respiration, suggesting that their mitochondria were better able to regulate oxidative stress. It is also possible that the increase in ATP production was related to the improvement in the ability of the mitochondria to handle oxidative stress. Since mitochondrial function is very intricately linked to inflammation and oxidative stress, the changes in mitochondrial function measured may have been related to changes in oxidative stress and inflammation, as has been suggested in other studies [51, 52]. Notably, Nrf2 expression is decreased in ASD [53] and is likely to be an important regulator of such “oxinflammation” [54, 55], and sulforaphane is an effective inducer of Nrf2 signaling [56, 57].

There are important differences in components of mitochondrial function in subgroups of ASD, notably in children with distinct histories of neurodevelopmental regression that may confound analysis of responses to treatments [21, 51]. We previously reported that children with ASD and developmental regression typically have *increased* maximal respiratory capacity at baseline (although also increased sensitivity to oxidative stress) [21]. However, we found relatively *decreased* levels with SF, whereas levels *increased* in children without developmental regression. These important differences may be a few among many that underlie the heterogeneity of ASD and emphasize the importance of delineating clinical subgroups and accounting for differential responses in therapeutic trials.

## Limitations

All clinical trials in children with ASD have inherent limitations due to clinical heterogeneity, cyclic variability in behaviors, sleep, feeding and bowel function (among others), and anxiety associated with adaptation to the intervention itself and necessary clinical visits. This study was no exception, and many of these factors limited the statistical significance of our clinical assessments. Biomarker data from our pilot and main studies helped to establish dosing and turnover of SF as well as cellular effects of SF. Clinical effects of GR + myrosinase as the source of SF were more difficult to ascertain in children than those in our previous trial of a SF-rich preparation in young men with ASD [6].

## Conclusions

We found that SF was safe, but based upon the OACIS, our primary clinical outcome measure, its effects were not significant. Effect size estimates showed small improvements with SF, though not significant, for the general level of autism on the OACIS-I scale. There was significant improvement on one of the secondary measures (ABC) but not on the SRS-2. Improvements in sociability and communication were observed on the SRS-2, as well as irritability, stereotypy, hyperactivity, and inappropriate speech on the ABC with a non-randomized analysis of the length of exposure. SF also had significant positive effects on oxidative stress, cytoprotective markers and cytokines, as well as mitochondrial function. These were promising findings that require further investigation of both the clinical effects and mechanisms of action of SF.

## Supplementary Information

**Additional file 1**. **Additional Files: Table S1(A–D).** Methods. **Table S2.** Characteristics of children who completed all visits vs children who dropped out of the study after 15 weeks**. Table S3.** OACIS-I scores *not including severely affected subjects*. **Table S4.** Descriptive analysis for raw total SRS-2 scores and subscale scores over all visits. **Table S5.** SRS-2 score means at each visit by intervention group, sulforaphane (SF) versus placebo (PL) (change from baseline). **Table S6.** SRS-2 washout effect (30–36 weeks) sulforaphane (SF) versus placebo (PL). **Table S7.** Descriptive analysis for total ABC scores and subscale scores**. Table S8.** ABC score means at each visit by intervention group, sulforaphane (SF) versus placebo (PL) (change from baseline). **Table S9.** ABC washout effect (30–36 weeks) sulforaphane (SF) versus placebo. **Table S10.** Length of exposure by fever response: SRS-2. **Table S11.** Length of sulforaphane (SF) exposure analysis by fever response: ABC. **Table S12.** Length of sulforaphane (SF) exposure by regression: SRS-2. **Table S13.** Length of sulforaphane (SF) exposure analysis by regression: ABC. **Table S14.** Mixed models accounting for treatment effect, visit and subject-specific random effect: SRS-2 total score and all subscales. **Table S15.** Mixed models accounting for treatment effect, visit and subject-specific random effect: ABC total score and all subscales. **Table S16.** Glutathione (GSH) variable means at each visit by intervention group, sulforaphane (SF) versus placebo (PL). **Table S17.** Descriptive analysis for glutathione (GSH) for total sample. **Table S18.** Univariate regression (ß) coefficients for sulforaphane (SF) compared to placebo (PL) for biomarkers (change from baseline). **Table S19.** Mean biomarker gene expression at 15 weeks of sulforaphane (SF) exposure (both groups) by regression (change from baseline).

## Data Availability

The datasets used and/or analyzed during this study are available herein or from the corresponding author on reasonable request.

## Abbreviations

ABC: Aberrant Behavior Checklist
ADOS-2: Autism Diagnostic Observation Schedule-2nd Edition
AKR1C1: Aldo–keto reductase family 1 member C1
ASD: Autism spectrum disorder
BMI: Body mass index
COX-2: Cyclooxygenase-2
DBPC: Double-blind, placebo-controlled
DTC: Dithiocarbamates
DSMB: Data Safety Monitoring Board
GR: Glucoraphanin
GSH: Glutathione
GSH: Glutathione
fGSH: Free reduced glutathione
tGSH: Total glutathione
GSSG: Oxidized glutathione disulfide
HO-1: Heme oxygenase 1
HSP27, HSP70: Heat shock proteins 27 and 70
IL-1β: Interleukin 1 beta
IL-6: Interleukin 6
ITC: Isothiocyanates
NQO1: NAD(P)H:quinone oxidoreductase-1
Nrf2: Nuclear factor erythroid 2-related factor 2
NF-κB: Nuclear factor-κB
OACIS-I: Ohio Autism Clinical Impressions Scale (or clinical global impression)—improvement
OACIS-S: Ohio Autism Clinical Impressions Scale (or clinical global impression)—severity
PBMC: Peripheral blood mononuclear cells
PBS: Phosphate buffered saline
PL: Placebo
SF: Sulforaphane
SRS-2: Social Responsiveness Scale-2
TNF-α: Tumor necrosis factor alpha
UMass: University of Massachusetts
xCT (SLC7A11): Cystine/glutamate antiporter encoded by the *SLC7A11* gene
