# Correction to: Randomized controlled trial of sulforaphane and metabolite discovery in children with Autism Spectrum Disorder

**DOI:** 10.1186/s13229-021-00451-9

**Published:** 2021-06-16

**Authors:** Andrew W. Zimmerman, Kanwaljit Singh, Susan L. Connors, Hua Liu, Anita A. Panjwani, Li‑Ching Lee, Eileen Diggins, Ann Foley, Stepan Melnyk, Indrapal N. Singh, S. Jill James, Richard E. Frye, Jed W. Fahey

**Affiliations:** 1 Departments of Pediatrics, Neurology and Psychiatry, University of Massachusetts Medical School, 55 N. Lake Ave., Worcester, MA 01655 USA; 2 Department of Pharmacology and Molecular Sciences, and The Cullman Chemoprotection Center, Johns Hopkins University School of Medicine, 725 N. Wolfe St., Baltimore, MD 21205 USA; 3 Department of Psychiatry and Behavioral Sciences, and iMIND Hopkins, Johns Hopkins University School of Medicine, 600 N. Wolfe St., Baltimore, MD 21287 USA; 4 Department of Psychological Sciences, Purdue University, 703 3rd St., West Lafayette, IN 47907 USA; 5 Department of Epidemiology, Johns Hopkins Bloomberg School of Public Health, 615 N. Wolfe St., Baltimore, MD 21205 USA; 6 Department of Pediatrics, University of Arkansas for Medical Sciences, 4301 W. Markham St., Little Rock, AR 72205 USA; 7 Barrow Neurologic Institute at Phoenix Children’s Hospital and Department of Child Health, University of Arizona College of Medicine – Phoenix, 475 N. 5th St., Phoenix, AZ 85004 USA; 8 Department of Medicine, Division of Clinical Pharmacology, Johns Hopkins University School of Medicine, 600 N. Wolfe St., Baltimore, MD 21287 USA

## Correction to: Molecular Autism (2021) 12:38 https://doi.org/10.1186/s13229-021-00447-5

Following publication of the original article [1], the authors pointed out that the part of the text in Methods part was incorrectly stipulated and should read as follows:

We were unable to produce or obtain SF-rich preparations for this study like those used in our previous study [6]. Based on prior testing of alternative preparations [25–27], we used commercially available crushable tablets containing broccoli seed and sprout extracts as a source of GR (equivalent to 34 µmol GR calculated to yield at least ~ 15 µmol SF) and active myrosinase enzyme. Tablets commercially produced as Avmacol® were provided by Nutramax Laboratories, Inc., Edgewood, MD. Placebo tablets, identical in size and similar in appearance to the active tablets, contained microcrystalline cellulose, red, yellow and black coloring and magnesium stearate, and were produced by Dr. Stephen Hoag, University of Maryland Department of Pharmacy.
