# Supplementary material for: Randomized controlled trial of sulforaphane and metabolite discovery in children with Autism Spectrum Disorder
Source: Mol Autism. 2021 May 25;12:38. doi: 10.1186/s13229-021-00447-5 (PMC8146218; doi:10.1186/s13229-021-00447-5)
Supplement: Supplementary file 1 — Additional file 1. Additional Files: Table S1(A–D). Methods. Table S2. Characteristics of children who completed all visits vs children who dropped out of the study after 15 weeks. Table S3. OACIS-I scores not including severely affected subjects. Table S4. Descriptive analysis for raw total SRS-2 scores and subscale scores over all visits. Table S5. SRS-2 score means at each visit by intervention group, sulforaphane (SF) versus placebo (PL) (change from baseline). Table S6. SRS-2 washout effect (30–36 weeks) sulforaphane (SF) versus placebo (PL). Table S7. Descriptive analysis for total ABC scores and subscale scores. Table S8. ABC score means at each visit by intervention group, sulforaphane (SF) versus placebo (PL) (change from baseline). Table S9. ABC washout effect (30–36 weeks) sulforaphane (SF) versus placebo. Table S10. Length of exposure by fever response: SRS-2. Table S11. Length of sulforaphane (SF) exposure analysis by fever response: ABC. Table S12. Length of sulforaphane (SF) exposure by regression: SRS-2. Table S13. Length of sulforaphane (SF) exposure analysis by regression: ABC. Table S14. Mixed models accounting for treatment effect, visit and subject-specific random effect: SRS-2 total score and all subscales. Table S15. Mixed models accounting for treatment effect, visit and subject-specific random effect: ABC total score and all subscales. Table S16. Glutathione (GSH) variable means at each visit by intervention group, sulforaphane (SF) versus placebo (PL). Table S17. Descriptive analysis for glutathione (GSH) for total sample. Table S18. Univariate regression (ß) coefficients for sulforaphane (SF) compared to placebo (PL) for biomarkers (change from baseline). Table S19. Mean biomarker gene expression at 15 weeks of sulforaphane (SF) exposure (both groups) by regression (change from baseline). [file 13229_2021_447_MOESM1_ESM.docx]

Zimmerman AW et al: RANDOMIZED CONTROLLED TRIAL OF SULFORAPHANE AND METABOLITE DISCOVERY IN CHILDREN WITH AUTISM SPECTRUM DISORDER

**Supporting Information** (Tables S1 – S19)

| Table S1(A-D). Methods   1. Sequences of real-time PCR primers | | |
| --- | --- | --- |
| Primers |  | Sequences |
| NQO1 | forward | 5'-CAG CTC ACC GAG AGC CTA GT-3' |
|  | reverse | 5'-GAG TGA GCC AGT ACG ATC AGT G-3' |
| HO-1 | forward | 5'-GGG TGA TAG AAG AGG CCA AGA-3' |
|  | reverse | 5'-AGC TCC TGC AAC TCC TCA AA-3' |
| xCT | forward | 5'-GCCCAAGGGGAGACACAAAA-3' |
|  | reverse | 5'-TGTGCGACTCATAGAATAACTGC-3' |
| HSP70 | forward | 5'-ATG AGT ATA GCG ACC GCT GC-3' |
|  | reverse | 5'-TCC TTG GAC TGT GTT CTT TGC-3' |
| HSP27 | forward | 5'-TCC CTG GAT GTC AAC CAC TTC-3' |
|  | reverse | 5'-TCT CCA CCA CGC CAT CCT-3' |
| COX-2 | forward | 5'-CAG CAC TTC ACG CAT CAG TTT T-3' |
|  | reverse | 5'-CCA GCC CGT TGG TGA AAG-3' |
| TNF-α | forward | 5'-ATC TTC TCG AAC CCC GAG TGA-3' |
|  | reverse | 5'-CGG TTC AGC CAC TGG AGC T-3' |
| IL-6 | forward | 5'-CGA GCC CAC CGG GAA CGA AA-3' |
|  | reverse | 5'-GGA CCG AAG GCG CTT GTG GAG-3' |
| IL-1β | forward | 5'-CAC GCT CCG GGA CTC ACA GC-3' |
|  | reverse | 5'-GGA GAA CAC CAC TTG TTG CTC CA-3' |
| GAPDH | forward | 5'-TGG TAT CGT GGA AGG ACT CA-3' |
|  | reverse | 5'-GGG CCA TCG ACA GTC TTC-3' |

1. Sample preparation for measurement of plasma total glutathione (tGSH)

To 200 μL of plasma 50 μL of 1.43 M sodium borohydride solution was added for reduction of the disulfide bonds. The solution was incubated at 40 ⁰C for 30 min following protein precipitation with 250 μL of 10% meta-phosphoric acid, and incubated on ice for 10 min. After centrifugation at 18,000 x g for 15 min at 4 ⁰C, 20 μL of the supernatant was injected into the HPLC system (30).

1. Sample preparation for measurement of plasma free reduced glutathione (fGSH) and oxidized glutathione disulfide (fGSSG)

To 200 μL of plasma 250 μL ice cold 10% meta-phosphoric acid was added and the sample incubated for 10 min on ice for protein precipitation with further centrifugation and injection to the HPLC system. The analyses were accomplished using HPLC-ECD and a reverse phase C_18_ column (5 μm; 4.6 x 150 mm, MCM, Inc., Tokyo, Japan). All plasma metabolites were quantified using a model 5200A Coulochem II detector (ESA, Inc., Chelmsford, MA). The concentrations of plasma metabolites were calculated from peak areas and standard calibration curves using HPLC software (30).

1. Measurement of mitochondrial function

Bioenergetic data were obtained from PBMCs using a state-of-the-art Seahorse 96 XF Analyzer (Seahorse Bioscience, Inc., North Billerica, MA) at three time points: before and after Phase 1 and after Phase 2. For detailed procedure see (21). Assay measurement curves were reviewed individually and plate wells with obvious assay failure were excluded from further analysis. Several key measurements can be derived from assay measurements, including ATP-Linked Respiration, Proton Leak Respiration, Maximal Respiratory Capacity and Reserve Capacity.

Table S2. Characteristics of children who completed all visits vs children who dropped out of the study after 15 weeks

| *Characteristic* | *Completed*  *(n=40)* | *Dropped Out*  *(n=5)* | *P-value^†^* |
| --- | --- | --- | --- |
| *Age (years), mean (SD)* | *7.3 (2.8)* | *6.8 (1.9)* | *0.73* |
| *Male sex, n (%)* | *35 (87.5)* | *5 (100.0)* | *0.41* |
| *Race, n (%)* |  |  | *0.32* |
| *White* | *29 (72.5)* | *3 (60.0)* |  |
| *Other^‡^* | *11 (27.5)* | *2 (40.0)* |  |
| *BMI (kg/m^2^), mean (SD)* | *16.9 (3.2)* | *18.0 (5.0)* | *0.48* |
| *Fever responder, n(%)* | *18 (45.0)* | *2 (40.0)* | *0.83* |
| *Regression, n(%)* | *14 (35.0)* | *2 (40.0)* | *0.83* |
| *Concomitant medications or therapy, n(%)* | *7 (17.5)* | *0 (0.0)* | *0.31* |
| *Baseline SRS-2 total raw score, mean (SD)* | *118.1 (21.8)* | *105.8 (21.8)* | *0.24* |
| *Baseline ABC total score, mean (SD)* | *69.5 (27.1)* | *45.4 (27.5)* | *0.07* |
| *Baseline OACIS-S general level of ASD* |  |  | *0.42^‡^* |
| *Mild/moderate n(%)* | *11 (27.5)* | *3 (60.0)* |  |
| *Marked, n(%)* | *18 (45.0)* | *1 (20.0)* |  |
| *Severe, n(%)* | *11 (27.5)* | *1 (20.0)* |  |
| *Total ADOS-2 Score, mean (SD)* | *7.8 (1.6)* | *7.0 (1.2)* | *0.36* |
| *ADOS-2 Social Affect Score, mean (SD)* | *12.8 (3.6)* | *9.8 (5.2)* | *0.12* |
| *ADOS-2 Repetitive Behavior Score, mean (SD)* | *5.3 (1.6)* | *6 (1.6)* | *0.39* |

BMI: body mass index, SRS-2: social responsiveness scale 2, ABC: aberrant behavior

checklist, OACIS-S: Ohio Autism Clinical Impressions Scale (or clinical global impression) – severity; ASD: autism spectrum disorder; ADOS-2: Autism Diagnostic Observation Schedule-2^nd^ Edition.

^†^Fisher’s exact chi-square for binary or categorical variables; t-test for continuous variables

^‡^Other race includes Asian, mixed, or unknown

Table S3. OACIS-I scores mean unit change from baseline (week 0) at each visit by intervention group and univariate linear regression of SF compared to placebo on CGI-I scores not including severely ill subjects

|  | **Paired T-test**^†^ | | | | | | | | | | **Effect Size**^‡^ |
| --- | --- | --- | --- | --- | --- | --- | --- | --- | --- | --- | --- |
| OACIS-I subscale | SF group N | | SF group Mean (SD) | | PL group N | | PL group Mean (SD) | | | p-value | Cohen’s d  (95% CI) |
| Total CGI-I |  | |  | |  | |  | | |  |  |
| Week 7 | 9 | | 0.56 (0.88) | | 9 | | 0.22 (0.44) | | | 0.347 | 0.48 (-0.50, 1.43) |
| Week 15 | 9 | | 0.56 (0.73) | | 9 | | 0.22 (0.44) | | | 0.195 | 0.55 (-0.27, 1.35) |
| Week 22 | 8 | | 0.88 (0.83) | | 8 | | 0.75 (0.71) | | | 0.785 | 0.16 (-0.96, 1.28) |
| Week 30 | 8 | | 0.69 (0.80) | | 8 | | 0.81 (0.84) | | | 0.785 | -0.15 (-1.20, 0.91) |
| Week 36 | 7 | | 0.29 (0.95) | | 7 | | 0.29 (0.49) | | | 1.000 | 0.00 (-0.98, 0.98) |
| Aberrant Abnormal Behaviors | |  | |  | |  | |  |  | |  |
| Week 7 | | 9 | | 0.11 (0.33) | | 9 | | 0.22 (0.44) | 0.594 | | -0.28 (-1.29, 0.74) |
| Week 15 | | 9 | | 0.11 (0.33) | | 9 | | 0.11 (0.60) | 1.000 | | 0.00 (-0.95, 0.95) |
| Week 22 | | 8 | | 0.25 (0.46) | | 8 | | 0.50 (0.93) | 0.517 | | -0.34 (-1.33, 0.67) |
| Week 30 | | 8 | | 0.38 (0.74) | | 8 | | 0.69 (0.96) | 0.529 | | -0.36 (-1.44, 0.74) |
| Week 36 | | 7 | | -0.14 (0.69) | | 7 | | 0.29 (0.49) | 0.078 | | -0.72 (-1.47, 0.08) |
| Social Interaction Severity |  | |  | |  | |  | | |  |  |
| Week 7 | 9 | | 0.56 (0.88) | | 9 | | 0.33 (0.71) | | | 0.594 | 0.28 (-0.72, 1.26) |
| Week 15 | 9 | | 0.56 (0.88) | | 9 | | 0.33 (0.50) | | | 0.447 | 0.31 (-0.47, 1.07) |
| Week 22 | 8 | | 1.00 (0.52) | | 8 | | 1.38 (0.93) | | | 0.197 | -0.50 (-1.22, 0.25) |
| Week 30 | 8 | | 1.00 (0.93) | | 8 | | 1.38 (0.52) | | | 0.285 | -0.50 (-1.37, 0.40) |
| Week 36 | 7 | | 0.29 (0.76) | | 7 | | 0.64 (0.63) | | | 0.182 | -0.51 (-1.22, 0.23) |
| Verbal Communication |  | |  | |  | |  | | |  |  |
| Week 7 | 9 | | 0.44 (0.73) | | 9 | | 0.44 (0.53) | | | 1.000 | 0.00 (-1.03, 1.03) |
| Week 15 | 9 | | 0.67 (0.87) | | 9 | | 0.72 (0.75) | | | 0.855 | -0.07 (-0.78, 0.64) |
| Week 22 | 8 | | 0.88 (1.13) | | 8 | | 1.25 (0.71) | | | 0.549 | -0.40 (-1.64, 0.87) |
| Week 30 | 8 | | 0.75 (0.89) | | 8 | | 1.19 (0.84) | | | 0.351 | -0.51 (-1.51, 0.54) |
| Week 36 | 7 | | 0.29 (0.49) | | 7 | | 0.86 (0.90) | | | 0.172 | -0.79 (-1.85, 0.33) |
| Hyperactivity Inattention |  | |  | |  | |  | | |  |  |
| Week 7 | 9 | | 0.33 (0.50) | | 9 | | 0.33 (0.50) | | | 1.000 | 0.00 (-0.92, 0.92) |
| Week 15 | 9 | | 0.44 (0.53) | | 9 | | 0.56 (0.88) | | | 0.760 | -0.15 (-1.10, 0.80) |
| Week 22 | 8 | | 0.75 (0.71) | | 8 | | 1.00 (0.93) | | | 0.626 | -0.30 (-1.47, 0.89) |
| Week 30 | 8 | | 0.63 (0.74) | | 8 | | 1.13 (1.13) | | | 0.407 | -0.52 (-1.70, 0.69) |
| Week 36 | 7 | | -0.14 (0.69) | | 7 | | 0.43 (0.53) | | | 0.172 | -0.93 (-2.17, 0.38) |

OACIS-I: Ohio Autism Clinical Impressions Scale (or clinical global impression) – improvement

^†^Paired t-test matched for sex and fever response

^‡^Standardized mean difference for matched pairs

Table S4. Descriptive analysis for raw total SRS-2 scores and sub-scale scores over all visits

| Raw SRS variable | N | Mean (SD) | Median |
| --- | --- | --- | --- |
| Total score |  |  |  |
| Visit 0 | 45 | 116.71 (21.91) | 118 |
| Visit 7 | 43 | 111.56 (23.19) | 108 |
| Visit 15 | 38 | 105.66 (22.58) | 107.5 |
| Visit 22 | 38 | 102.37 (22.63) | 99.5 |
| Visit 30 | 31 | 102.55 (26.67) | 97 |
| Visit 36 | 17 | 108.47 (23.46) | 113 |
| Social Awareness |  |  |  |
| Visit 0 | 45 | 14.80 (3.38) | 15 |
| Visit 7 | 43 | 14.65 (3.12) | 14 |
| Visit 15 | 38 | 14.00 (3.25) | 14 |
| Visit 22 | 38 | 13.37 (3.79) | 13 |
| Visit 30 | 31 | 13.84 (3.15) | 13 |
| Visit 36 | 17 | 13.47 (3.68) | 13 |
| Social Cognition |  |  |  |
| Visit 0 | 45 | 22.2 (4.61) | 23 |
| Visit 7 | 43 | 21.72 (4.41) | 23 |
| Visit 15 | 38 | 20.11 (4.96) | 21 |
| Visit 22 | 38 | 19.76 (4.86) | 18.5 |
| Visit 30 | 31 | 19.97 (5.19) | 20 |
| Visit 36 | 17 | 19.65 (4.95) | 20 |
| Social Communication |  |  |  |
| Visit 0 | 45 | 40.22 (8.13) | 41 |
| Visit 7 | 43 | 38.21 (8.94) | 37 |
| Visit 15 | 38 | 36.37 (8.9) | 35 |
| Visit 22 | 38 | 35.89 (8.91) | 36 |
| Visit 30 | 31 | 36.13 (9.54) | 35 |
| Visit 36 | 17 | 37.18 (9.66) | 38 |
| Social Motivation |  |  |  |
| Visit 0 | 45 | 16.82 (5.16) | 17 |
| Visit 7 | 43 | 15.98 (5.27) | 16 |
| Visit 15 | 38 | 14.61 (5.23) | 14 |
| Visit 22 | 38 | 14.21 (4.91) | 14.5 |
| Visit 30 | 31 | 13.61 (4.92) | 12 |
| Visit 36 | 17 | 15.24 (5.70) | 14 |
| RRB^†^ |  |  |  |
| Visit 0 | 45 | 22.67 (6.02) | 23 |
| Visit 7 | 43 | 21.00 (5.86) | 20 |
| Visit 15 | 38 | 20.58 (5.56) | 21 |
| Visit 22 | 38 | 19.13 (5.92) | 18 |
| Visit 30 | 31 | 19.00 (7.78) | 16 |
| Visit 36 | 17 | 21.94 (4.81) | 22 |
| SCI^‡^ |  |  |  |
| Visit 0 | 45 | 94.04 (17.37) | 95 |
| Visit 7 | 43 | 90.56 (18.79) | 88 |
| Visit 15 | 38 | 85.08 (18.98) | 85.5 |
| Visit 22 | 38 | 83.24 (18.21) | 81 |
| Visit 30 | 31 | 83.55 (19.99) | 81 |
| Visit 36 | 17 | 86.53 (20.08) | 87 |

SRS-2: Social Responsiveness Scale-2

^†^RRB: Restricted Interests & Repetitive Behavior

^‡^SCI: Social Communication & Interaction

Table S5. SRS-2 score means at each visit by intervention group, Sulforaphane (SF) vs. Placebo (PL) (change from baseline)

|  | **Paired T-test**^†^ | | | | | **Effect Size**^‡^ |
| --- | --- | --- | --- | --- | --- | --- |
| SRS-2 variable | SF group N | SF group  Mean (SD) | PL group N | Placebo group Mean (SD) | P-value | Cohen’s d  (95% CI) |
| Total score |  |  |  |  |  |  |
| Baseline | 17 | 119.53 (28.30) | 17 | 116.59 (18.04) | 0.77 | 0.12 (-0.68, 0.92) |
| Week 7 | 15 | 1.47 (17.06) | 15 | -9.73 (15.98) | 0.09 | 0.68 (-0.11, 1.45) |
| Week 15 | 12 | -14.00 (18.49) | 12 | -9.75 (16.22) | 0.58 | -0.24 (-1.08, 0.60) |
| Week 22 | 13 | -14.92 (16.60) | 13 | -14.54 (15.60) | 0.96 | -0.02 (-0.87, 0.82) |
| Week 30 | 10 | -14.20 (16.67) | 10 | -19.90 (23.13) | 0.41 | 0.28 (-0.38, 0.93) |
| Week 36 | 4 | -7.00 (10.61) | 4 | -4.75 (14.34) | 0.79 | -0.18 (-1.38, 1.05) |
| Social Awareness |  |  |  |  |  |  |
| Baseline | 17 | 16.18 (3.52) | 17 | 14.00 (3.10) | 0.13 | 0.66 (-0.19, 1.48) |
| Week 7 | 15 | -0.13 (2.47) | 15 | -0.13 (1.77) | 1.00 | 0.00 (-0.67, 0.67) |
| Week 15 | 12 | -2.25 (2.66) | 12 | -1.00 (2.52) | 0.34 | -0.48 (-1.44, 0.49) |
| Week 22 | 13 | -1.92 (2.02) | 13 | -1.23 (2.20) | 0.49 | -0.33 (-1.22, 0.58) |
| Week 30 | 10 | -2.20 (1.81) | 10 | -1.30 (3.27) | 0.45 | -0.34 (-1.18, 0.52) |
| Week 36 | 4 | -2.75 (1.71) | 4 | -2.25 (3.30) | 0.83 | -0.19 (-2.05, 1.70) |
| Social Cognition |  |  |  |  |  |  |
| Baseline | 17 | 21.71 (6.56) | 17 | 22.47 (3.30) | 0.72 | -0.15 (-0.92, 0.64) |
| Week 7 | 15 | 1.20 (5.58) | 15 | -2.00 (3.07) | 0.10 | 0.71 (-0.13, 1.53) |
| Week 15 | 12 | -2.42 (4.50) | 12 | -1.58 (3.55) | 0.65 | -0.21 (-1.07, 0.67) |
| Week 22 | 13 | -1.85 (6.16) | 13 | -2.69 (4.81) | 0.71 | 0.15 (-0.69, 0.99) |
| Week 30 | 10 | -2.50 (5.56) | 10 | -2.80 (4.02) | 0.83 | 0.06 (-0.48, 0.60) |
| Week 36 | 4 | -3.75 (4.79) | 4 | -2.25 (2.36) | 0.68 | -0.40 (-2.12, 1.39) |
| Social Communication | |  |  |  |  |  |
| Baseline | 17 | 41.88 (9.95) | 17 | 40.29 (6.64) | 0.65 | 0.19 (-0.61, 0.98) |
| Week 7 | 15 | 0.00 (5.62) | 15 | -3.47 (5.90) | 0.11 | 0.60 (-0.13, 1.32) |
| Week 15 | 12 | -4.25 (4.77) | 12 | -3.92 (7.27) | 0.89 | -0.09 (-0.81, 0.63) |
| Week 22 | 13 | -4.92 (5.62) | 13 | -4.38 (5.79) | 0.80 | -0.05 (-0.78, 0.67) |
| Week 30 | 10 | -3.60 (4.38) | 10 | -6.70 (8.60) | 0.17 | 0.45 (-0.19, 1.07) |
| Week 36 | 4 | -1.00 (5.50) | 4 | -1.25 (4.90) | 0.89 | 0.05 (-0.56, 0.65) |
| Social Motivation |  |  |  |  |  |  |
| Baseline | 17 | 16.65 (6.05) | 17 | 17.06 (4.67) | 0.83 | -0.08 (-0.77, 0.62) |
| Week 7 | 15 | 0.73 (3.59) | 15 | -1.47 (4.13) | 0.13 | 0.57 (-0.11, 1.23) |
| Week 15 | 12 | -0.83 (3.66) | 12 | -2.50 (3.94) | 0.31 | 0.44 (-0.58, 1.44) |
| Week 22 | 13 | -2.00 (5.48) | 13 | -2.38 (4.99) | 0.86 | 0.07 (-0.65, 0.80) |
| Week 30 | 10 | -1.80 (3.46) | 10 | -2.80 (5.82) | 0.65 | 0.21 (-0.71, 1.11) |
| Week 36 | 4 | 2.75 (2.75) | 4 | 2.50 (5.74) | 0.95 | 0.06 (-1.43, 1.53) |
| RRB^§^ |  |  |  |  |  |  |
| Baseline | 17 | 23.12 (7.03) | 17 | 22.76 (5.25) | 0.89 | 0.06 (-0.63, 0.75) |
| Week 7 | 15 | -0.33 (6.10) | 15 | -2.67 (5.17) | 0.26 | 0.41 (-0.30, 1.11) |
| Week 15 | 12 | -4.25 (6.63) | 12 | -0.75 (4.79) | 0.14 | -0.61 (-1.44, 0.26) |
| Week 22 | 13 | -4.23 (4.59) | 13 | -3.85 (4.96) | 0.84 | -0.08 (-0.89, 0.73) |
| Week 30 | 10 | -4.10 (6.19) | 10 | -6.30 (6.57) | 0.41 | 0.34 (-0.56, 1.23) |
| Week 36 | 4 | -2.25 (3.20) | 4 | -1.5 (5.80) | 0.86 | -0.16 (-1.75, 1.46) |
| SCI^¶^ |  |  |  |  |  |  |
| Baseline | 17 | 96.41 (22.27) | 17 | 93.82 (13.84) | 0.74 | 0.14 (-0.62, 0.90) |
| Week 7 | 15 | 1.80 (12.18) | 15 | -7.07 (11.68) | 0.08 | 0.74 (-0.01, 1.48) |
| Week 15 | 12 | -9.75 (12.34) | 12 | -9.00 (12.20) | 0.89 | -0.06 (-0.79, 0.67) |
| Week 22 | 13 | -10.69 (13.54) | 13 | -10.69 (11.66) | 1.00 | 0.00 (-0.78, 0.78) |
| Week 30 | 10 | -10.10 (11.59) | 10 | -13.60 (17.30) | 0.45 | 0.24 (-0.56, 1.03) |
| Week 36 | 4 | -4.75 (9.98) | 4 | -3.25 (10.21) | 0.84 | -0.15 (-1.77, 1.50) |

SRS-2: Social Responsiveness Scale-2

^†^Paired t-test, matched for sex and fever response; Baseline values are raw scores

^‡^Standardized mean difference for matched pairs

^§^RRB: Restricted Interests & Repetitive Behavior

^¶^SCI: Social Communication & Interaction

Table S6. SRS-2 Wash-out effect (30-36 weeks) Sulforaphane (SF) vs. Placebo (PL)

| SRS variable | SF group  F-statistic  (n=6) | SF group  P-value | PL group  F-statistic  (n=10) | PL group  P-value |
| --- | --- | --- | --- | --- |
| Total score | 2.47 | 0.18 | 0.06 | 0.81 |
| Social Awareness | 0.04 | 0.84 | 0.17 | 0.69 |
| Social Cognition | 0.13 | 0.73 | 0.57 | 0.47 |
| Social Communication | 1.09 | 0.34 | 0.75 | 0.41 |
| Social Motivation | 17.10 | **0.01** | 0.01 | 0.93 |
| RRB^†^ | 4.57 | 0.09 | 1.14 | 0.31 |
| SCI^‡^ | 1.95 | 0.22 | 0.66 | 0.44 |

SRS-2: social responsiveness scale 2

^†^RRB: Restricted Interests & Repetitive Behavior

^‡^SCI: Social Communication & Interaction

Table S7. Descriptive analysis for total ABC scores and sub-scale scores

| ABC variable | N | Mean (SD) | Median |
| --- | --- | --- | --- |
| Total score |  |  |  |
| Visit 0 | 45 | 66.78 (27.86) | 65.00 |
| Visit 7 | 43 | 58.53 (24.38) | 56.00 |
| Visit 15 | 39 | 55.51 (21.46) | 53.00 |
| Visit 22 | 35 | 46.94 (22.70) | 43.00 |
| Visit 30 | 32 | 50.56 (26.58) | 45.00 |
| Visit 36 | 17 | 62.94 (25.58) | 61.00 |
| Sqrt Lethargy^†^ |  |  |  |
| Visit 0 | 45 | 3.43 (1.14) | 3.46 |
| Visit 7 | 43 | 3.27 (1.29) | 3.32 |
| Visit 15 | 39 | 3.00 (1.35) | 3.00 |
| Visit 22 | 35 | 2.71 (1.31) | 2.65 |
| Visit 30 | 32 | 2.71 (1.49) | 2.65 |
| Visit 36 | 17 | 3.32 (1.33) | 3.32 |
| Sqrt Irritability |  |  |  |
| Visit 0 | 45 | 3.73 (1.35) | 3.87 |
| Visit 7 | 43 | 3.53 (1.10) | 3.46 |
| Visit 15 | 39 | 3.44 (1.20) | 3.46 |
| Visit 22 | 35 | 3.08 (1.02) | 3.32 |
| Visit 30 | 32 | 3.16 (1.28) | 3.31 |
| Visit 36 | 17 | 3.67 (1.04) | 3.61 |
| Sqrt Stereotypy |  |  |  |
| Visit 0 | 45 | 2.74 (1.01) | 2.65 |
| Visit 7 | 43 | 2.48 (1.13) | 2.65 |
| Visit 15 | 39 | 2.60 (0.91) | 2.65 |
| Visit 22 | 35 | 2.51 (0.92) | 2.45 |
| Visit 30 | 32 | 2.50 (1.14) | 2.55 |
| Visit 36 | 17 | 2.97 (0.96) | 3.32 |
| Hyperactivity |  |  |  |
| Visit 0 | 45 | 24.09 (9.67) | 23.00 |
| Visit 7 | 43 | 20.63 (9.76) | 20.00 |
| Visit 15 | 39 | 19.62 (8.29) | 20.00 |
| Visit 22 | 35 | 16.51 (9.79) | 15.00 |
| Visit 30 | 32 | 18.22 (9.34) | 16.50 |
| Visit 36 | 17 | 20.71 (9.30) | 22.00 |
| Inappropriate Speech |  |  |  |
| Visit 0 | 45 | 5.47 (3.28) | 5.00 |
| Visit 7 | 43 | 4.56 (2.48) | 5.00 |
| Visit 15 | 39 | 4.33 (3.16) | 4.00 |
| Visit 22 | 35 | 3.77 (2.67) | 4.00 |
| Visit 30 | 31 | 3.94 (2.34) | 4.00 |
| Visit 36 | 17 | 5.35 (3.37) | 5.00 |

ABC: Aberrant Behavior Checklist

^†^Sqrt: square root

Table S8. ABC score means at each visit by intervention group, Sulforaphane (SF) vs. Placebo (PL) (change from baseline)

|  | **Paired T-test**^†^ | | | | | | **Effect Size**^‡^ |
| --- | --- | --- | --- | --- | --- | --- | --- |
| ABC variable | SF group N | SF group Mean (SD) | PL group N | PL group Mean (SD) | P-value | | Cohen’s d  (95% CI) |
| Total score |  |  |  |  |  | |  |
| Baseline | 17 | 74.53 (25.32) | 17 | 61.47 (25.15) | | 0.20 | 0.39 (-0.30, 1.07) |
| Week 7 | 15 | -2.00 (18.68) | 15 | -11.00 (16.99) | 0.13 | | 0.44 (-0.16, 1.02) |
| Week 15 | 12 | -19.50 (18.47) | 12 | -2.92 (16.10) | **0.02** | | **-0.96 (-1.73, -0.15)** |
| Week 22 | 10 | -25.60 (24.41) | 10 | -21.30 (11.45) | 0.51 | | -0.23 (-0.87, 0.43) |
| Week 30 | 11 | -12.91 (18.15) | 11 | -11.73 (25.20) | 0.91 | | -0.05 (-0.97, 0.86) |
| Week 36 | 4 | -4.00 (27.23) | 4 | 1.00 (14.97) | 0.82 | | -0.23 (-2.08, 1.66) |
| Sqrt Lethargy^§^ |  |  |  |  |  | |  |
| Baseline | 17 | 3.71 (1.25) | 17 | 3.22 (1.16) | | 0.31 | 0.41 (-0.37, 1.17) |
| Week 7 | 15 | 0.30 (0.73) | 15 | -0.38 (0.89) | **0.02** | | **0.83 (0.11, 1.53)** |
| Week 15 | 12 | -0.19 (0.57) | 12 | -0.33 (1.06) | 0.63 | | 0.16 (-0.48, 0.80) |
| Week 22 | 10 | -0.55 (0.78) | 10 | -0.78 (0.50) | 0.43 | | 0.36 (-0.52, 1.23) |
| Week 30 | 11 | -0.31 (0.66) | 11 | -0.68 (1.57) | 0.50 | | 0.31 (-0.57, 1.17) |
| Week 36 | 4 | 0.23 (0.65) | 4 | 0.58 (1.01) | 0.70 | | -0.34 (-2.53, 1.93) |
| Sqrt Irritability |  |  |  |  |  | |  |
| Baseline | 17 | 3.99 (1.39) | 17 | 3.48 (1.38) | | 0.32 | 0.37 (-0.35, 1.07) |
| Week 7 | 15 | -0.04 (0.95) | 15 | -0.37 (0.79) | 0.28 | | 0.37 (-0.29, 1.01) |
| Week 15 | 12 | -0.86 (0.89) | 12 | 0.00 (0.82) | 0.07 | | -1.00 (-2.05, 0.09) |
| Week 22 | 10 | -0.98 (0.99) | 10 | -0.82 (0.66) | 0.67 | | -0.19 (-1.02, 0.65) |
| Week 30 | 11 | -0.60 (1.10) | 11 | -0.43 (1.10) | 0.71 | | -0.16 (-1.02, 0.71) |
| Week 36 | 4 | -0.17 (1.65) | 4 | -0.17 (0.89) | 0.99 | | 0.00 (-1.49, 1.49) |
| Sqrt Stereotypy |  |  |  |  |  | |  |
| Baseline | 17 | 2.74 (1.02) | 17 | 2.79 (1.16) | | 0.89 | -0.05 (-0.73, 0.64) |
| Week 7 | 15 | -0.30 (1.07) | 15 | -0.27 (0.62) | 0.92 | | -0.03 (-0.79, 0.72) |
| Week 15 | 12 | -0.15 (0.63) | 12 | -0.13 (0.39) | 0.92 | | -0.04 (-0.70, 0.63) |
| Week 22 | 10 | -0.08 (0.51) | 10 | -0.78 (0.39) | **<0.01** | | **1.54 (0.49, 2.55)** |
| Week 30 | 11 | -0.06 (0.76) | 11 | -0.57 (0.97) | 0.23 | | 0.59 (-0.35, 1.49) |
| Week 36 | 4 | 0.06 (0.82) | 4 | -0.30 (0.25) | 0.51 | | 0.59 (-1.08, 2.18) |
| Hyperactivity |  |  |  |  |  | |  |
| Baseline | 17 | 26.53 (9.58) | 17 | 21.71 (9.12) | | 0.18 | 0.52 (-0.24, 1.26) |
| Week 7 | 15 | -2.07 (7.13) | 15 | -4.13 (4.85) | 0.25 | | 0.35 (-0.22, 0.91) |
| Week 15 | 12 | -8.75 (10.12) | 12 | -0.92 (6.60) | **0.04** | | **-1.67 (-2.70, -0.59)** |
| Week 22 | 10 | -11.00 (11.15) | 10 | -6.20 (5.43) | 0.18 | | **-1.22 (-2.18, -0.22)** |
| Week 30 | 11 | -4.64 (5.20) | 10 | -3.91 (9.28) | 0.84 | | -0.09 (-1.01, 0.83) |
| Week 36 | 4 | -3.50 (5.00) | 4 | -2.25 (6.18) | 0.83 | | -0.22 (-2.03, 1.62) |
| Inappropriate Speech |  |  |  |  |  | |  |
| Baseline | 17 | 6.59 (3.47) | 17 | 5.12 (2.76) | 0.26 | | 0.47 (-0.34, 1.26) |
| Week 7 | 15 | -1.40 (3.56) | 15 | -0.40 (2.20) | 0.37 | | -0.34 (-1.10, 0.43) |
| Week 15 | 12 | -2.83 (3.71) | 12 | 0.05 (2.93) | **<0.01** | | **-0.85 (-1.49, -0.19)** |
| Week 22 | 10 | -3.10 (4.01) | 10 | -1.10 (2.08) | 0.09 | | -0.63 (-1.32, 0.10) |
| Week 30 | 10 | -2.10 (2.28) | 10 | -0.10 (1.73) | **0.049** | | **-0.99 (-1.89, -0.05)** |
| Week 36 | 4 | 0.50 (2.65) | 4 | 1.25 (1.71) | 0.75 | | -0.34 (-2.21, 1.59) |

ABC: Aberrant Behavior Checklist

^†^Paired t-test, matched for sex and fever response

^‡^Standardized mean difference for matched pairs

^§^Sqrt: square root; Baseline values are raw scores (or sqrt raw scores for transformed subscales)

Table S9. ABC Wash-out effect (30-36 weeks) Sulforaphane (SF) vs. Placebo (PL)

|  | SF group | | PL group | |
| --- | --- | --- | --- | --- |
| ABC variable | F-statistic  (n=6) | P-value | F-statistic (n=10) | P-value |
| Total score | 33.03 | **0.002** | 1.33 | 0.278 |
| Lethargy^‡^ | 4.68 | 0.083 | 1.32 | 0.280 |
| Irritability^‡^ | 5.00 | 0.076 | 0.29 | 0.600 |
| Stereotypy^‡^ | 3.34 | 0.127 | 0.81 | 0.392 |
| Hyperactivity | 2.62 | 0.166 | 1.46 | 0.258 |
| Inappropriate Speech | 3.46 | 0.122 | 0.67 | 0.438 |

ABC: Aberrant Behavior Checklist

^‡^Square root transformed

Table S10. Length of exposure by fever response: SRS-2^†^

|  |  | Fever responders | | Non-fever responders | | |
| --- | --- | --- | --- | --- | --- | --- |
| SRS variable | N | F-statistic | P-value | N | F-statistic | P-value |
| Total score |  |  |  |  |  |  |
| 7 weeks | 15 | 1.70 | 0.213 | 23 | 0.15 | 0.704 |
| 15 weeks | 15 | 2.93 | 0.089 | 19 | 8.84 | **0.002** |
| 22 weeks | 10 | 2.02 | 0.200 | 9 | 5.89 | **0.032** |
| 30 weeks^‡^ | - | - | - | 9 | 8.38 | **0.019** |
| Social Awareness |  |  |  |  |  |  |
| 7 weeks | 15 | 1.09 | 0.315 | 23 | 0.52 | 0.478 |
| 15 weeks | 15 | 1.20 | 0.333 | 19 | 1.16 | **0.002** |
| 22 weeks | 10 | 2.18 | 0.179 | 9 | 5.28 | **0.040** |
| 30 weeks | - | - | - | 9 | 3.31 | 0.111 |
| Social Cognition |  |  |  |  |  |  |
| 7 weeks | 15 | 0.00 | 0.960 | 23 | 0.08 | 0.780 |
| 15 weeks | 15 | 2.28 | 0.142 | 19 | 2.27 | 0.133 |
| 22 weeks | 10 | 1.24 | 0.366 | 9 | 1.60 | 0.286 |
| 30 weeks | - | - | - | 9 | 3.53 | **0.010** |
| Social Communication |  |  |  |  |  |  |
| 7 weeks | 15 | 1.74 | 0.209 | 23 | 0.23 | 0.638 |
| 15 weeks | 15 | 1.97 | 0.178 | 19 | 10.83 | **0.001** |
| 22 weeks | 10 | 2.47 | 0.147 | 9 | 4.96 | **0.046** |
| 30 weeks | - | - | - | 9 | 3.42 | 0.105 |
| Social Motivation |  |  |  |  |  |  |
| 7 weeks | 15 | 1.42 | 0.254 | 23 | 4.04 | **0.057** |
| 15 weeks | 15 | 1.01 | 0.393 | 19 | 5.02 | **0.019** |
| 22 weeks | 10 | 1.60 | 0.273 | 9 | 2.19 | 0.190 |
| 30 weeks | - | - | - | 9 | 3.01 | 0.129 |
| RRB^§^ |  |  |  |  |  |  |
| 7 weeks | 15 | 3.12 | 0.099 | 23 | 1.36 | 0.256 |
| 15 weeks | 15 | 3.74 | **0.052** | 19 | 6.16 | **0.010** |
| 22 weeks | 10 | 1.73 | 0.248 | 9 | 6.23 | **0.028** |
| 30 weeks | - | - | - | 9 | 3.89 | 0.084 |
| SCI^¶^ |  |  |  |  |  |  |
| 7 weeks | 15 | 1.15 | 0.302 | 23 | 0.00 | 0.948 |
| 15 weeks | 15 | 2.38 | 0.131 | 19 | 8.84 | **0.002** |
| 22 weeks | 10 | 1.90 | 0.219 | 9 | 6.10 | **0.030** |
| 30 weeks | - | - | - | 9 | 12.20 | **0.009** |

^†^SRS-2: Social Responsiveness Scale-2; MANOVA test for repeated measures (compared to baseline at 0 weeks)

^‡^30 weeks for fever responders not available due to collinearity between repeated weeks

^§^RRB: Restricted Interests & Repetitive Behavior; ^¶^SCI: Social Communication and Interaction

Table S11. Length of sulforaphane exposure analysis by fever response: ABC^†^

|  |  | | Fever responders | | Non-fever responders | | | |  |
| --- | --- | --- | --- | --- | --- | --- | --- | --- | --- |
| ABC variable | | N | F-statistic | P-value | | N | F-statistic | P-value | |
| Total score | |  |  |  | |  |  |  | |
| 7 weeks | | 16 | 4.91 | **0.043** | | 22 | 4.63 | **0.043** | |
| 15 weeks | | 16 | 5.35 | **0.019** | | 18 | 8.40 | **0.003** | |
| 22 weeks | | 10 | 2.45 | 0.149 | | 6 | 1.86 | 0.312 | |
| 30 weeks | | 6 | 1.36 | 0.466 | | 6 | 1.43 | 0.452 | |
| Sqrt Lethargy | |  |  |  | |  |  |  | |
| 7 weeks | | 16 | 0.72 | 0.408 | | 22 | 0.79 | 0.386 | |
| 15 weeks | | 16 | 2.27 | 0.140 | | 18 | 2.59 | 0.106 | |
| 22 weeks | | 10 | 1.95 | 0.211 | | 6 | 1.33 | 0.411 | |
| 30 weeks | | 6 | 0.58 | 0.714 | | 6 | 0.71 | 0.658 | |
| Sqrt Irritability | |  |  |  | |  |  |  | |
| 7 weeks | | 16 | 2.99 | 0.104 | | 22 | 2.92 | 0.120 | |
| 15 weeks | | 16 | 7.01 | 0.008 | | 18 | 3.34 | 0.061 | |
| 22 weeks | | 10 | 2.60 | 0.134 | | 6 | 1.33 | 0.411 | |
| 30 weeks | | 6 | 1.00 | 0.555 | | 6 | 0.84 | 0.607 | |
| Sqrt Stereotypy | |  |  |  | |  |  |  | |
| 7 weeks | | 16 | 3.94 | 0.066 | | 22 | 1.37 | 0.255 | |
| 15 weeks | | 16 | 2.45 | 0.123 | | 18 | 4.08 | **0.037** | |
| 22 weeks | | 10 | 0.34 | 0.797 | | 6 | 1.26 | 0.427 | |
| 30 weeks | | 6 | 0.21 | 0.910 | | 6 | 1.61 | 0.418 | |
| Hyperactivity | |  |  |  | |  |  |  | |
| 7 weeks | | 16 | 7.17 | **0.017** | | 22 | 3.01 | 0.098 | |
| 15 weeks | | 16 | 3.98 | **0.043** | | 18 | 6.64 | **0.008** | |
| 22 weeks | | 10 | 2.32 | 0.162 | | 6 | 6.01 | 0.088 | |
| 30 weeks | | 6 | 4.93 | 0.176 | | 6 | 3.13 | 0.257 | |
| Inappropriate Speech | |  |  |  | |  |  |  | |
| 7 weeks | | 16 | 2.07 | 0.171 | | 22 | 6.49 | **0.019** | |
| 15 weeks | | 16 | 1.63 | 0.232 | | 17 | 2.80 | **0.093** | |
| 22 weeks | | 10 | 1.67 | 0.261 | | 6 | 2.44 | 0.242 | |
| 30 weeks | | 6 | 2.07 | 0.351 | | 6 | 1.82 | 0.385 | |

^†^ABC: Aberrant Behavior Checklist; MANOVA test for repeated measures (compared to baseline at 0 weeks)

Table S12. Length of sulforaphane exposure by regression: SRS-2^†^

|  |  | Regression | | No regression | | |
| --- | --- | --- | --- | --- | --- | --- |
| SRS variable | N | F-statistic | P-value | N | F-statistic | P-value |
| Total score |  |  |  |  |  |  |
| 7 weeks | 13 | 0.00 | 0.984 | 25 | 1.09 | 0.306 |
| 15 weeks | 11 | 3.49 | 0.076 | 23 | 6.67 | **0.006** |
| 22 weeks | 9 | 2.32 | 0.175 | 10 | 3.18 | 0.094 |
| 30 weeks | 6 | 4.39 | 0.194 | 8 | 13.76 | **0.013** |
| Social Awareness |  |  |  |  |  |  |
| 7 weeks | 13 | 1.99 | 0.184 | 23 | 0.26 | 0.617 |
| 15 weeks | 11 | 3.89 | 0.061 | 19 | 4.72 | **0.020** |
| 22 weeks | 9 | 1.72 | 0.262 | 9 | 8.83 | **0.009** |
| 30 weeks | 6 | 5.83 | 0.152 | 9 | 4.98 | 0.075 |
| Social Cognition |  |  |  |  |  |  |
| 7 weeks | 13 | 0.94 | 0.351 | 23 | 1.25 | 0.274 |
| 15 weeks | 11 | 1.12 | 0.368 | 19 | 4.37 | **0.026** |
| 22 weeks | 9 | 2.74 | 0.135 | 9 | 1.87 | 0.223 |
| 30 weeks | 6 | 1.61 | 0.418 | 9 | 5.14 | 0.071 |
| Social Communication |  |  |  |  |  |  |
| 7 weeks | 13 | 0.07 | 0.798 | 23 | 1.72 | 0.202 |
| 15 weeks | 11 | 3.75 | 0.065 | 19 | 6.74 | **0.006** |
| 22 weeks | 9 | 1.98 | 0.218 | 9 | 2.80 | 0.118 |
| 30 weeks | 6 | 1.18 | 0.508 | 9 | 6.55 | **0.048** |
| Social Motivation |  |  |  |  |  |  |
| 7 weeks | 13 | 0.00 | 0.958 | 23 | 0.03 | 0.856 |
| 15 weeks | 11 | 1.50 | 0.274 | 19 | 2.72 | 0.089 |
| 22 weeks | 9 | 1.64 | 0.277 | 9 | 1.71 | 0.252 |
| 30 weeks | 6 | 5.00 | 0.174 | 9 | 1.50 | 0.353 |
| ^‡^RRB |  |  |  |  |  |  |
| 7 weeks | 13 | 0.88 | 0.368 | 23 | 3.79 | 0.063 |
| 15 weeks | 11 | 2.61 | 0.128 | 19 | 8.12 | **0.002** |
| 22 weeks | 9 | 0.94 | 0.476 | 9 | 4.80 | **0.040** |
| 30 weeks | 6 | 3.05 | 0.262 | 9 | 3.42 | 0.130 |
| ^¶^SCI |  |  |  |  |  |  |
| 7 weeks | 13 | 0.00 | 0.984 | 23 | 1.09 | 0.306 |
| 15 weeks | 11 | 3.49 | 0.076 | 19 | 6.67 | **0.006** |
| 22 weeks | 9 | 2.32 | 0.175 | 9 | 3.18 | 0.094 |
| 30 weeks | 6 | 4.39 | 0.194 | 9 | 13.76 | **0.013** |

^†^SRS-2: Social Responsiveness Scale 2; MANOVA test for repeated measures (compared to baseline at 0 weeks)

^‡^RRB: Restricted Interests & Repetitive Behavior

^¶^SCI: Social Communication and Interaction

Table S13. Length of sulforaphane exposure analysis by regression: ABC^†^

|  |  | | Regression | | No regression | | | |  |
| --- | --- | --- | --- | --- | --- | --- | --- | --- | --- |
| ABC variable | | N | F-statistic | P-value | | N | F-statistic | P-value | |
| Total score | |  |  |  | |  |  |  | |
| 7 weeks | | 13 | 2.37 | 0.150 | | 25 | 7.08 | **0.014** | |
| 15 weeks | | 11 | 8.58 | **0.008** | | 23 | 6.97 | **0.005** | |
| 22 weeks | | 8 | 2.58 | 0.166 | | 8 | 3.98 | 0.086 | |
| 30 weeks | | 6 | 2.15 | 0.342 | | 6 | 40.76 | **0.024** | |
| ^‡^Sqrt Lethargy | |  |  |  | |  |  |  | |
| 7 weeks | | 13 | 0.55 | 0.474 | | 25 | 2.51 | 0.126 | |
| 15 weeks | | 11 | 3.60 | 0.071 | | 23 | 3.16 | 0.063 | |
| 22 weeks | | 8 | 1.06 | 0.445 | | 8 | 8.03 | **0.023** | |
| 30 weeks | | 6 | 0.77 | 0.634 | | 6 | 11.24 | 0.083 | |
| Sqrt Irritability | |  |  |  | |  |  |  | |
| 7 weeks | | 13 | 2.58 | 0.134 | | 25 | 3.39 | 0.078 | |
| 15 weeks | | 11 | 4.41 | **0.046** | | 23 | 5.07 | **0.016** | |
| 22 weeks | | 8 | 2.82 | 0.147 | | 8 | 6.34 | **0.037** | |
| 30 weeks | | 6 | 2.98 | 0.267 | | 6 | 2.98 | 0.267 | |
| Sqrt Stereotypy | |  |  |  | |  |  |  | |
| 7 weeks | | 13 | 0.56 | 0.470 | | 25 | 4.60 | **0.042** | |
| 15 weeks | | 11 | 2.62 | 0.127 | | 23 | 4.90 | **0.018** | |
| 22 weeks | | 8 | 1.50 | 0.264 | | 8 | 0.61 | 0.640 | |
| 30 weeks | | 6 | 0.80 | 0.621 | | 6 | 0.08 | 0.982 | |
| Hyperactivity | |  |  |  | |  |  |  | |
| 7 weeks | | 13 | 2.04 | 0.179 | | 25 | 7.92 | **0.010** | |
| 15 weeks | | 11 | 5.64 | **0.026** | | 23 | 6.58 | **0.006** | |
| 22 weeks | | 8 | 3.17 | 0.123 | | 8 | 4.18 | 0.079 | |
| 30 weeks | | 6 | 1.60 | 0.419 | | 6 | 3.21 | 0.251 | |
| Inappropriate Speech | |  |  |  | |  |  |  | |
| 7 weeks | | 13 | 4.09 | 0.066 | | 25 | 4.19 | **0.052** | |
| 15 weeks | | 11 | 1.87 | 0.210 | | 23 | 2.06 | 0.153 | |
| 22 weeks | | 8 | 0.79 | 0.551 | | 8 | 2.49 | 0.175 | |
| 30 weeks | | 6 | 0.96 | 0.567 | | 6 | 0.93 | 0.576 | |

^†^ABC: Aberrant Behavior Checklist; MANOVA test for repeated measures (compared to baseline at 0 weeks)

^‡^Sqrt: Square root

Table S14. Mixed Models: SRS-2^†^

|  | Total Score | | Social  Awareness | | Social  Cognition | | Social  Communication | | Social  Motivation | | Restricted Interests &  Repetitive behaviors | | | SCI | |
| --- | --- | --- | --- | --- | --- | --- | --- | --- | --- | --- | --- | --- | --- | --- | --- |
|  | β | P-value | β | P-value | β | P-value | β | P-value | β | P-value | β | P-value | β | | P-value |
| Model 1 |  |  |  |  |  |  |  |  |  |  |  |  |  | |  |
| Treatment |  |  |  |  |  |  |  |  |  |  |  |  |  | |  |
| Placebo | (ref) |  | (ref) |  | (ref) |  | (ref) |  | (ref) |  | (ref) |  | (ref) | |  |
| SF | 4.21 | 0.22 | 1.53 | **<0.01** | 0.02 | 0.98 | 1.70 | 0.47 | -0.30 | 0.71 | 1.18 | 0.21 | 3.00 | | 0.28 |
| Week |  |  |  |  |  |  |  |  |  |  |  |  |  | |  |
| 0 | (ref) |  | (ref) |  | (ref) |  | (ref) |  | (ref) |  | (ref) |  | (ref) | |  |
| 7 | -5.09 | 0.16 | -0.18 | 0.75 | -0.46 | 0.55 | -2.08 | **0.02** | -0.83 | 0.34 | -1.62 | 0.10 | -3.47 | | 0.23 |
| 15 | -10.23 | **0.02** | -0.82 | 0.20 | -1.88 | **0.03** | -3.76 | **<0.001** | -2.06 | **0.04** | -1.83 | 0.11 | -8.42 | | **0.01** |
| 22 | -13.19 | **0.01** | -1.49 | **0.04** | -2.34 | **0.02** | -4.30 | **<0.001** | -2.21 | **0.05** | -3.16 | **0.01** | -10.08 | | **0.01** |
| 30 | -16.08 | **0.01** | -1.23 | 0.16 | -2.47 | **0.04** | -5.34 | **<0.001** | -3.08 | **0.02** | -3.85 | **0.02** | -12.24 | | **0.01** |
| Model 2 |  |  |  |  |  |  |  |  |  |  |  |  |  | |  |
| Treatment |  |  |  |  |  |  |  |  |  |  |  |  |  | |  |
| Placebo | (ref) |  | (ref) |  | (ref) |  | (ref) |  | (ref) |  | (ref) |  | (ref) | |  |
| SF | -2.12 | 0.82 | 0.18 | 0.87 | -1.64 | 0.34 | 0.66 | 0.85 | -0.27 | 0.88 | -0.28 | 0.91 | -1.53 | | 0.83 |
| Week |  |  |  |  |  |  |  |  |  |  |  |  |  | |  |
| 30 | (ref) |  | (ref) |  | (ref) |  | (ref) |  | (ref) |  | (ref) |  | (ref) | |  |
| 36 | 3.26 | 0.47 | -0.26 | 0.73 | -0.45 | 0.72 | 0.40 | 0.82 | 1.40 | 0.12 | 2.58 | 0.09 | 1.00 | | 0.79 |
| Model 3 |  |  |  |  |  |  |  |  |  |  |  |  |  | |  |
| Treatment |  |  |  |  |  |  |  |  |  |  |  |  |  | |  |
| Placebo | (ref) |  | (ref) |  | (ref) |  | (ref) |  | (ref) |  | (ref) |  | (ref) | |  |
| SF | 6.63 | 0.25 | 1.94 | **0.03** | -0.36 | 0.77 | 3.13 | 0.15 | 0.70 | 0.61 | 0.82 | 0.58 | 5.42 | | 0.25 |
| Week |  |  |  |  |  |  |  |  |  |  |  |  |  | |  |
| 0 | (ref) |  | (ref) |  | (ref) |  | (ref) |  | (ref) |  | (ref) |  | (ref) | |  |
| 36 | -7.73 | 0.23 | -1.16 | 0.25 | -2.56 | 0.07 | -1.86 | 0.45 | -1.67 | 0.29 | -0.53 | 0.75 | -7.24 | | 0.18 |

^†^SRS-2: Social Responsiveness Scale-2; Model 1: testing treatment effect; Model 2: testing wash-out effect; Model 3: testing change from baseline

Table S15: Mixed Models: ABC^†^

|  | Total Score | | Sqrt Lethargy^‡^ | | Sqrt Irritability | | Sqrt Stereotypy | | Hyperactivity | | Inappropriate Speech | |
| --- | --- | --- | --- | --- | --- | --- | --- | --- | --- | --- | --- | --- |
|  | β | P-value | β | P-value | β | P-value | β | P-value | β | P-value | β | P-value |
| Model 1 |  |  |  |  |  |  |  |  |  |  |  |  |
| Treatment |  |  |  |  |  |  |  |  |  |  |  |  |
| Placebo | (ref) |  | (ref) |  | (ref) |  | (ref) |  | (ref) |  | (ref) |  |
| SF | 11.63 | **<0.01** | 0.41 | **0.03** | 0.35 | 0.06 | 0.06 | 0.73 | 4.04 | **0.01** | 0.77 | 0.09 |
| Week |  |  |  |  |  |  |  |  |  |  |  |  |
| 0 | (ref) |  | (ref) |  | (ref) |  | (ref) |  | (ref) |  | (ref) |  |
| 7 | -8.36 | **0.049** | -0.16 | 0.42 | -0.20 | 0.31 | -0.25 | 0.14 | -3.51 | **0.03** | -0.93 | 0.07 |
| 15 | -11.20 | **0.02** | -0.42 | 0.07 | -0.30 | 0.19 | -0.12 | 0.55 | -4.42 | **0.01** | -1.17 | **0.04** |
| 22 | -20.01 | **<0.001** | -0.68 | **0.01** | -0.69 | **0.01** | -0.27 | 0.24 | -7.33 | **<0.001** | -1.63 | **0.01** |
| 30 | -14.46 | **0.02** | -0.63 | 0.06 | -0.40 | 0.18 | -0.25 | 0.35 | -5.41 | **0.02** | -1.70 | **0.02** |
| Model 2 |  |  |  |  |  |  |  |  |  |  |  |  |
| Treatment |  |  |  |  |  |  |  |  |  |  |  |  |
| Placebo | (ref) |  | (ref) |  | (ref) |  | (ref) |  | (ref) |  | (ref) |  |
| SF | -2.50 | 0.78 | 0.29 | 0.54 | -0.33 | 0.41 | -0.26 | 0.49 | 1.13 | 0.73 | -1.76 | **0.04** |
| Week |  |  |  |  |  |  |  |  |  |  |  |  |
| 30 | (ref) |  | (ref) |  | (ref) |  | (ref) |  | (ref) |  | (ref) |  |
| 36 | 9.87 | **0.046** | 0.32 | 0.10 | 0.45 | 0.12 | 0.32 | 0.10 | 2.59 | 0.10 | 0.86 | **0.03** |
| Model 3 |  |  |  |  |  |  |  |  |  |  |  |  |
| Treatment |  |  |  |  |  |  |  |  |  |  |  |  |
| Placebo | (ref) |  | (ref) |  | (ref) |  | (ref) |  | (ref) |  | (ref) |  |
| SF | 11.19 | 0.11 | 0.57 | **0.05** | 0.24 | 0.47 | 0.07 | 0.78 | 3.95 | 0.11 | 1.18 | 0.17 |
| Week |  |  |  |  |  |  |  |  |  |  |  |  |
| 0 | (ref) |  | (ref) |  | (ref) |  | (ref) |  | (ref) |  | (ref) |  |
| 36 | -2.83 | 0.72 | -0.11 | 0.76 | **-0.01** | 0.98 | 0.26 | 0.36 | -3.06 | 0.26 | -0.02 | 0.98 |

^†^ABC: Aberrant Behavior Checklist; Model 1: testing treatment effect; Model 2: testing wash-out effect; Model 3: testing change from baseline

^‡^Sqrt: Square root

Table S16. Glutathione (GSH) variable means at each visit by intervention group,

Sulforaphane (SF) vs Placebo (PL)

| GSH biomarker | SF group N | SF group Mean (SD) | PL group N | PL group  Mean (SD) | P-value |
| --- | --- | --- | --- | --- | --- |
| Free reduced^†^ |  |  |  |  |  |
| 0 weeks | 16 | 0.52 (0.14) | 18 | 0.60 (0.16) | 0.180 |
| 15 weeks | 17 | 0.52 (0.16) | 19 | 0.59 (0.19) | 0.266 |
| 30 weeks | 19 | 0.58 (0.18) | 17 | 0.60 (0.14) | 0.700 |
| Free reduced  (NHS)^†‡^ |  |  |  |  |  |
| 0 weeks | 16 | 0.52 (0.14) | 18 | 0.60 (0.16) | 0.180 |
| 15 weeks | 17 | 0.52 (0.16) | 19 | 0.59 (0.19) | 0.266 |
| 30 weeks | 19 | 0.58 (0.18) | 17 | 0.60 (0.14) | 0.700 |
| Total* |  |  |  |  |  |
| 0 weeks | 16 | 1.79 (0.19) | 18 | 1.81 (0.15) | 0.740 |
| 15 weeks | 17 | 1.84 (0.13) | 18 | 1.85 (0.14) | 0.865 |
| 30 weeks | 19 | 1.90 (0.12) | 17 | 1.89 (0.11) | 0.845 |
| Total (NHS)^†‡^ |  |  |  |  |  |
| 0 weeks | 16 | 1.79 (0.19) | 18 | 1.81 (0.15) | 0.740 |
| 15 weeks | 17 | 1.84 (0.13) | 18 | 1.85 (0.14) | 0.865 |
| 30 weeks | 19 | 1.90 (0.12) | 17 | 1.89 (0.11) | 0.845 |
| Oxidized |  |  |  |  |  |
| 0 weeks | 22 | -1.81 (0.31) | 24 | -1.79 (0.23) | 0.736 |
| 15 weeks | 22 | -1.71 (0.24) | 23 | -1.75 (0.21) | 0.602 |
| 30 weeks | 22 | -1.64 (0.20) | 19 | -1.76 (0.23) | 0.071 |
| GSH:GSSG |  |  |  |  |  |
| 0 weeks | 22 | 12.11 (1.84) | 24 | 11.64 (1.84) | 0.392 |
| 15 weeks | 22 | 10.13 (1.75) | 23 | 11.23 (2.80) | 0.124 |
| 30 weeks | 22 | 9.83 (1.87) | 19 | 11.28 (1.78) | **0.015** |
| Total GSH:GSSG |  |  |  |  |  |
| 0 weeks | 22 | 43.51 (8.80) | 24 | 40.43 (5.94) | 0.168 |
| 15 weeks | 22 | 38.88 (6.15) | 23 | 40.22 (8.73) | 0.555 |
| 30 weeks | 22 | 36.97 (6.40) | 19 | 41.56 (6.73) | **0.031** |

^†^Natural log transformed values

^‡^NHS: Not including hemolyzed samples

Table S17. Descriptive analysis for glutathione (GSH) for total sample

| GSH biomarker | N | Mean (SD) | Median | |
| --- | --- | --- | --- | --- |
| Free reduced^†^ |  |  |  | |
| 0 weeks | 46 | 0.66 (0.24) | 0.61 | |
| 15 weeks | 45 | 0.62 (0.20) | 0.60 | |
| 30 weeks | 41 | 0.64 (0.20) | 0.56 | |
| Free reduced^†‡^ |  |  |  | |
| 0 weeks | 34 | 0.56 (0.16) | 0.56 | |
| 15 weeks | 36 | 0.56 (0.18) | 0.56 | |
| 30 weeks | 36 | 0.59 (0.16) | 0.55 | |
| Total^†^ |  |  |  | |
| 0 weeks | 46 | 1.92 (0.27) | 1.86 | |
| 15 weeks | 45 | 1.93 (0.21) | 1.91 | |
| 30 weeks | 41 | 1.95 (0.20) | 1.88 | |
| Total^†‡^ |  |  |  |  |
| 0 weeks | 34 | 1.80 (0.17) | 1.78 |  |
| 15 weeks | 35 | 1.84 (0.13) | 1.85 |  |
| 30 weeks | 36 | 1.89 (0.11) | 1.87 |  |
| Oxidized^†^ |  |  |  | |
| 0 weeks | 46 | -1.80 (0.27) | -1.84 | |
| 15 weeks | 45 | -1.73 (0.22) | -1.76 | |
| 30 weeks | 41 | -1.70 (0.22) | -1.71 | |
| GSH:GSSG |  |  |  | |
| 0 weeks | 46 | 11.87 (1.83) | 11.74 | |
| 15 weeks | 45 | 10.69 (2.39) | 10.17 | |
| 30 weeks | 41 | 10.50 (1.95) | 10.18 | |
| Total GSH:GSSG |  |  |  | |
| 0 weeks | 46 | 41.91 (7.48) | 41.03 | |
| 15 weeks | 45 | 39.57 (7.52) | 38.91 | |
| 30 weeks | 41 | 39.10 (6.87) | 37.66 | |

^†^Natural log transformed values

^‡^NHS: Not including hemolyzed samples

Table S18. Univariate regression (β) coefficients for Sulforaphane (SF)

compared to Placebo (PL) for biomarkers (change from baseline)

|  | β coefficient | 95% CI | P value |
| --- | --- | --- | --- |
| ln NQO1^†^ |  |  |  |
| Week 7 | -0.34 | (-1.02, 0.33) | 0.310 |
| Week 15 | -0.45 | (-1.27, 0.36) | 0.267 |
| Week 22 | 0.25 | (-0.60, 1.11) | 0.550 |
| Week 30 | -0.51 | (-1.41, 0.40) | 0.262 |
| Week 36 | 0.20 | (-0.80, 1.20) | 0.678 |
| ln xCT |  |  |  |
| Week 7 | 0.01 | (-0.59, 0.61) | 0.961 |
| Week 15 | 0.37 | (-0.17, 0.92) | 0.173 |
| Week 22 | -0.34 | (-1.10, 0.41) | 0.361 |
| Week 30 | 0.12 | (-0.69, 0.92) | 0.770 |
| Week 36 | -0.25 | (-1.16, 0.65) | 0.570 |
| ln HO-1 |  |  |  |
| Week 7 | -0.50 | (-1.13, 0.14) | 0.121 |
| Week 15 | -0.84 | (-1.50, -0.19) | **0.013** |
| Week 22 | 0.21 | (-0.45, 0.87) | 0.521 |
| Week 30 | -0.29 | (-1.04, 0.47) | 0.444 |
| Week 36 | 0.04 | (-0.60, 0.69) | 0.890 |
| ln HSP70 |  |  |  |
| Week 7 | -0.41 | (-0.90, 0.07) | 0.091 |
| Week 15 | -0.78 | (-1.45, -0.10) | **0.025** |
| Week 22 | 0.21 | (-0.24, 0.65) | 0.350 |
| Week 30 | -0.11 | (-0.85, 0.63) | 0.765 |
| Week 36 | 0.25 | (-0.41, 0.91) | 0.436 |
| ln HSP27 |  |  |  |
| Week 7 | -0.04 | (-0.27, 0.18) | 0.693 |
| Week 15 | -0.31 | (-0.66, 0.04) | 0.078 |
| Week 22 | -0.02 | (-0.38, 0.35) | 0.926 |
| Week 30 | -0.19 | (-0.51, 0.13) | 0.229 |
| Week 36 | 0.02 | (-0.47, 0.51) | 0.937 |
| ln IL-6 |  |  |  |
| Week 7 | -0.39 | (-0.94, 0.17) | 0.166 |
| Week 15 | -1.31 | (-2.22, -0.39) | **0.006** |
| Week 22 | -0.12 | (-0.97, 0.72) | 0.768 |
| Week 30 | -0.01 | (-0.87, 0.85) | 0.981 |
| Week 36 | 0.02 | (-0.89, 0.93) | 0.969 |
| ln IL-1β |  |  |  |
| Week 7 | -1.13 | (-2.11, -0.14) | **0.026** |
| Week 15 | -0.76 | (-1.88, 0.35) | 0.174 |
| Week 22 | -0.26 | (-1.36, 0.83) | 0.627 |
| Week 30 | -1.32 | (-2.55, -0.09) | **0.037** |
| Week 36 | -0.66 | (-2.55, 1.24) | 0.483 |
| ln COX-2 |  |  |  |
| Week 7 | 0.12 | (-0.30, 0.54) | 0.562 |
| Week 15 | 0.20 | (-0.25, 0.64) | 0.377 |
| Week 22 | -0.04 | (-0.49, 0.41) | 0.862 |
| Week 30 | -0.04 | (-0.51, 0.44) | 0.874 |
| Week 36 | -0.17 | (-0.78, 0.43) | 0.558 |
| ln TNF-α |  |  |  |
| Week 7 | -0.18 | (-0.62, 0.25) | 0.394 |
| Week 15 | -0.56 | (-0.98, -0.14) | **0.010** |
| Week 22 | -0.04 | (-0.67, 0.58) | 0.885 |
| Week 30 | -0.50 | (-1.13, 0.13) | 0.113 |
| Week 36 | 0.04 | (-0.70, 0.77) | 0.922 |

^†^ln: Natural log transformed values

Table S19. Mean biomarker gene expression at 15 weeks of sulforaphane exposure (both groups) by

developmental regression (change from baseline)^†^

|  | DevelopmentalRegression  Effect N | Regression  Effect  Mean (95% CI) | No Regression  Effect  N | No Regression  Effect  Mean (95% CI) | P value |
| --- | --- | --- | --- | --- | --- |
| NQO1 |  |  |  |  |  |
| Baseline | 16 | 9.86 (8.71, 11.01) | 26 | 9.40 (8.65, 10.15) | 0.467 |
| 15 weeks | 14 | 0.82 (0.03, 1.61) | 22 | 0.56 (-0.06, 1.18) | 0.591 |
| xCT |  |  |  |  |  |
| Baseline | 17 | 1.80 (1.00, 2.59) | 27 | 1.36 (0.89, 1.82) | 0.293 |
| 15 weeks | 14 | 0.10 (-0.64, 0.83) | 24 | 0.09 (-0.31, 0.48) | 0.979 |
| HO-1 |  |  |  |  |  |
| Baseline | 17 | 1.32 (0.71, 1.93) | 27 | 1.62 (1.09, 2.15) | 0.459 |
| 15 weeks | 14 | -0.18 (-0.82, 0.47) | 24 | -0.56 (-1.19, 0.06) | 0.404 |
| HSP70 |  |  |  |  |  |
| Baseline | 17 | 0.15 (-0.56, 0.87) | 27 | -0.10 (-0.59, 0.40) | 0.541 |
| 15 weeks | 14 | -0.06 (-0.40, 0.28) | 24 | -0.25 (-0.47, -0.03) | 0.296 |
| HSP27 |  |  |  |  |  |
| Baseline | 17 | 3.04 (2.48, 3.61) | 27 | 3.01 (2.62, 3.40) | 0.923 |
| 15 weeks | 14 | -0.20 (-0.84, 0.44) | 24 | -0.86 (-1.50, -0.22) | 0.163 |
| IL-6 |  |  |  |  |  |
| Baseline | 17 | 6.58 (5.62, 7.54) | 27 | 6.13 (5.37, 6.89) | 0.448 |
| 15 weeks | 14 | -0.87 (-1.46, -0.28) | 23 | -1.50 (-2.17, -0.83) | 0.185 |
| IL-1β |  |  |  |  |  |
| Baseline | 17 | 3.10 (1.53, 4.67) | 27 | 3.05 (1.94, 4.15) | 0.954 |
| 15 weeks | 14 | -0.25 (-1.18, 0.68) | 22 | -1.30 (-2.20, -0.40) | 0.113 |
| COX2 |  |  |  |  |  |
| Baseline | 17 | 0.33 (-0.63, 1.29) | 27 | 0.19 (-0.25, 0.63) | 0.747 |
| 15 weeks | 14 | -0.04 (-0.48, 0.41) | 24 | -0.24 (-0.41, -0.07) | 0.286 |
| TNFα |  |  |  |  |  |
| Baseline | 17 | 2.74 (2.18, 3.30) | 27 | 2.41 (1.87, 2.95) | 0.411 |
| 15 weeks | 14 | -0.48 (-0.89, -0.06) | 24 | -0.71 (-1.13, -0.29) | 0.441 |

^†^All biomarkers are natural log transformed values
